# Tunable Brownian magneto heat pump

**DOI:** 10.1038/s41598-022-17584-3

**Published:** 2022-08-04

**Authors:** Iman Abdoli, René Wittmann, Joseph Michael Brader, Jens-Uwe Sommer, Hartmut Löwen, Abhinav Sharma

**Affiliations:** 1 Institut Theorie der Polymere, Leibniz-Institut für Polymerforschung Dresden, 01069 Dresden, Germany; 2 Institut für Theoretische Physik, Technische Universität Dresden, 01069 Dresden, Germany; 3 Institut für Theoretische Physik II, Weiche Materie, Heinrich-Heine-Universität Düsseldorf, 40225 Düsseldorf, Germany; 4 Department de Physique, Université de Fribourg, CH-1700 Fribourg, Switzerland

**Keywords:** Magnetic properties and materials, Statistical physics, Thermodynamics

## Abstract

We propose a mesoscopic Brownian magneto heat pump made of a single charged Brownian particle that is steered by an external magnetic field. The particle is subjected to two thermal noises from two different heat sources. When confined, the particle performs gyrating motion around a potential energy minimum. We show that such a magneto-gyrator can be operated as both a heat engine and a refrigerator. The maximum power delivered by the engine and the performance of the refrigerator, namely the rate of heat transferred per unit external work, can be tuned and optimised by the applied magnetic field. Further tunability of the key properties of the engine, such as the direction of gyration and the torque exerted by the engine on the confining potential, is obtained by varying the strength and direction of the applied magnetic field. In principle, our predictions can be tested by experiments with colloidal particles and complex plasmas.

## Introduction

A heat pump is a device that transfers thermal energy between two heat baths. Recently, much attention has been paid to the study of heat pumps on colloidal and molecular scales^1–8^. Brownian heat pumps function as heat engines by converting thermal energy into mechanical work while pumping heat from hot to cold bath^9–11^. A Brownian heat engine generates power through the rectification of thermal fluctuations^12–15^. Onsager symmetry implies that, in linear response regime (i.e., close to equilibrium), a thermal Brownian engine can be operated as a refrigerator^9,16,17^ upon performance of external work which makes thermal current go against the temperature gradient^16,18^. Our current understanding of such micro/nano devices is based on stochastic thermodynamics, a powerful theoretical framework that extends the concepts of heat, work and entropy production to small systems dominated by thermal fluctuations^19,20^. Less than a decade ago, Blickle and Bechinger^21^ devised the first experimental realisation of a microscopic heat engine. They made a microscopic Stirling engine, where a single Brownian particle was subjected to a time-dependent optical trap and periodically coupled to different heat baths. Ever since, several nano-, micro-, and meso-scopic scale devices at a single atom^22^ or colloidal particle^23–28^ level have been experimentally realised.

**Figure 1:**
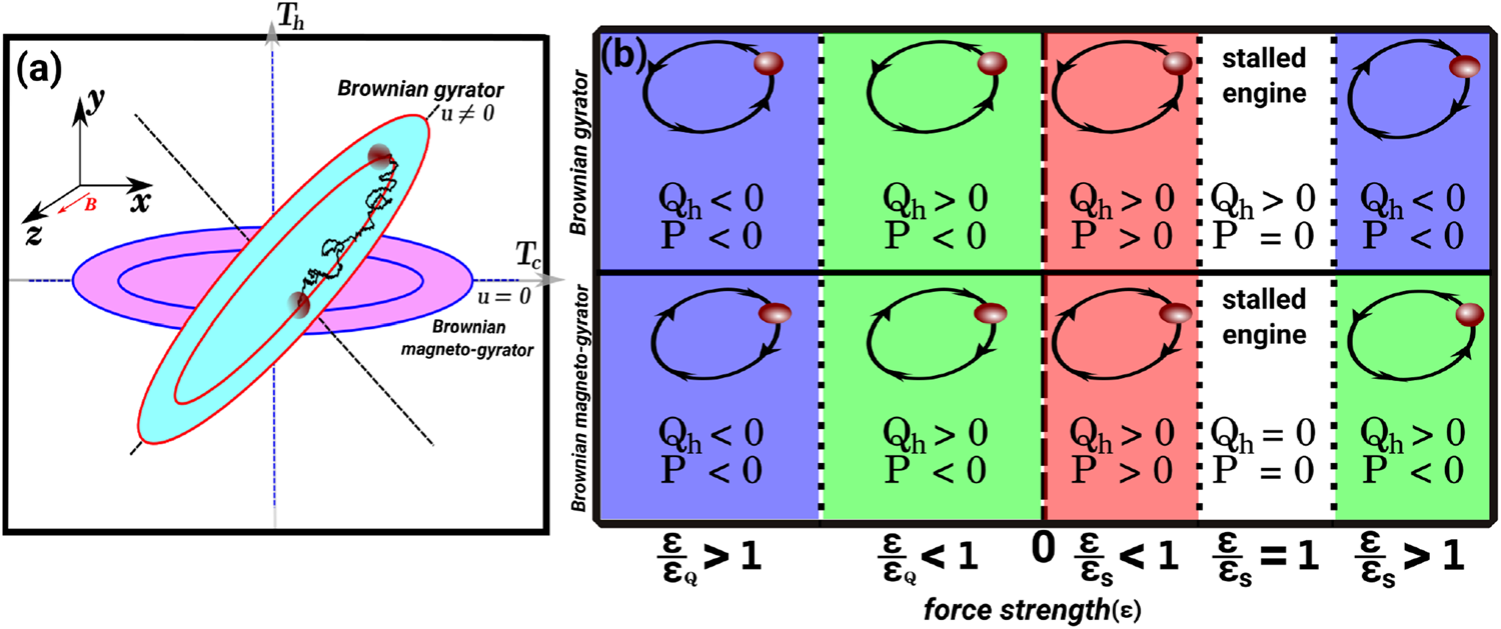
A single charged Brownian particle, steered by an external magnetic field *B* and trapped in a rotationally asymmetric potential, performs gyration when subjected to different thermal noises from cold, $T_c$, and hot, $T_h$, heat baths coupled to its *x* and *y* degrees of freedom, respectively. (**a**) schematically depicts the diffusion of the particle in a magnetic field in the *z* direction and under the influence of the harmonic potential $V(x,y)=k[(x^2+\alpha y^2)/2+uxy]$ with the parameters *k* and $\alpha$. Here *u* is the potential coupling parameter which correlates the spatial degrees of freedom. The generic scenario is considered when the principal axes of the potential, shown by dashed lines, are misaligned with the temperature axes, namely if $u\ne 0$. Note that this condition (i.e., $u\ne 0$) is only necessary for the system to operate as a Brownian gyrator in the absence of a magnetic field. In contrast to a Brownian gyrator, in a magneto-gyrator the magnetic field couples the spatial degrees of freedom of the particle such that the particle gyrates even for $u=0$. By considering the nonconservative force ${\varvec{F}}_{nc}=\ \varepsilon (-y, x)^\top$ where $\varepsilon$ is the strength of the force, the system can be operated as a refrigerator or a heat engine. (**b**) Schematic illustration of the operation of a Brownian gyrator (top) and a Brownian magneto-gyrator (bottom) with respect to the strength of the force. The directions of gyration are shown by arrows. The work extraction due to the nonconservative force is given as $P=-\langle {\varvec{F}}_{nc}^\top .{\dot{{\varvec{r}}}}\rangle$. The blue and red regions correspond to the operation of the systems as a refrigerator (by putting work into the systems or loading beyond stalling, $P<0$, to make thermal current go against the temperature gradient) and a heat engine (by loading the system to extract work, $P>0$), respectively. The white color shows the point where the engines are stalled (i.e., at the stall parameter $\varepsilon _s$). The green regions represent a range of $\varepsilon$ where the systems can be operated neither as a refrigerator nor as a heat engine. Note that by putting work into the systems, corresponding to $\varepsilon <0$, the heat flow changes its direction at $\varepsilon _Q$. In fact a magneto-gyrator under loading, corresponding to $\varepsilon >0$, can not perform cooling. The directions of gyration are shown with $u>0$ for a Brownian gyrator (i.e., in the absence of a magnetic field) and a positive magnetic field for a Brownian magneto-gyrator (i.e., $u=0$). Note that $Q_h>0$ and $Q_h<0$ are the heat out of and into the hot bath, respectively.

Tuning the performance of such microdevices is a highly desired property^9,11,28^. Here, we propose a novel way of tuning the properties via an external magnetic field. Our device is a tunable Brownian magneto heat pump made of a single charged Brownian particle subjected to Lorentz force due to a constant external magnetic field. The particle is simultaneously subjected to different thermal noises along its spatial degrees of freedom, which can be experimentally realised by applying a strongly fluctuating electric field to one direction mimicking the role of an additional temperature^29^. The particle performs gyrating motion around a potential energy minimum (see Fig. 1a). We show that such a magneto-gyrator can be operated as both a heat engine and a refrigerator (see Fig. 1b). The magneto-refrigerator is made by applying an external work to make heat energy go against the temperature gradient. The maximum power delivered by the engine and the performance of a magneto-refrigerator, namely the rate of heat transferred per unit external work, can be tuned and optimised by the applied magnetic field. Furthermore, the fundamental properties of the engine, such as the direction of gyration and the torque exerted by the engine on the confining potential can be tuned by varying the strength and direction of the applied magnetic field.

Microengines subjected to an external magnetic field have been studied in the past in the context of the broken-time reversal symmetry and its effect on the efficiency and power of such engines^30–34^. Recently, the effect of Lorentz force has been studied in active and passive colloidal systems which are dominated by overdamped dynamics^35–46^. We show in this work that the heat flow in a magneto-gyrator is governed by the momenta correlations which require the explicit knowledge of the mass of the particle. In the absence of a magnetic field, the momenta variables are decoupled and hence the heat flow ceases to exist. The momenta governed heat transfer was shown by Murashita and Esposito in Ref.^47^ where they considered the Brownian particle to be isotropically coupled to different thermostats. A magneto-gyrator thus presents an experimentally realisable system to measure heat currents governed by momenta. The momenta governed heat transfer in a magneto-gyrator is analogous to the phonon Hall effect which was first discovered in 2005 in a paramagnetic dielectric^48^. In that experimental work, Strohm, Rikken, and Wyder showed that in a paramagnetic dielectric subjected to a longitudinal temperature gradient, a magnetic field induces a transverse heat current perpendicular to the applied magnetic field and to the longitudinal temperature gradient. While in the phonon Hall effect the system is subjected to a spatial temperature gradient, a magneto-gyrator has its two degrees of freedom connected to heat baths at different temperature. Since then, the control and manipulation of energy, for instance in form of heat, in phononics have attracted much attention^49–51^. As in the phonon Hall effect, the rate of heat flow in the magneto-gyrator can be tuned by the external magnetic field.

Using values from the literature^29,52^, we show that a magneto-refrigerator with a coefficient of performance (ratio of heat extracted to the work done) greater than 2.0 and a heat engine with an efficiency of $\sim 0.15\eta _{c}$ at maximum power could be realised in dusty plasmas, where $\eta _{c}$ is the Carnot efficiency.

The paper continues as follows: we first introduce a Brownian magneto heat pump operating as a heat engine for which we investigate the torque, work, heat loss, and efficiency and their tunability. Next, we operate the pump as a magneto-refrigerator where we study the optimisation of its performance through the applied magnetic field.

## Results

### Brownian magneto heat pump

**Figure 2:**
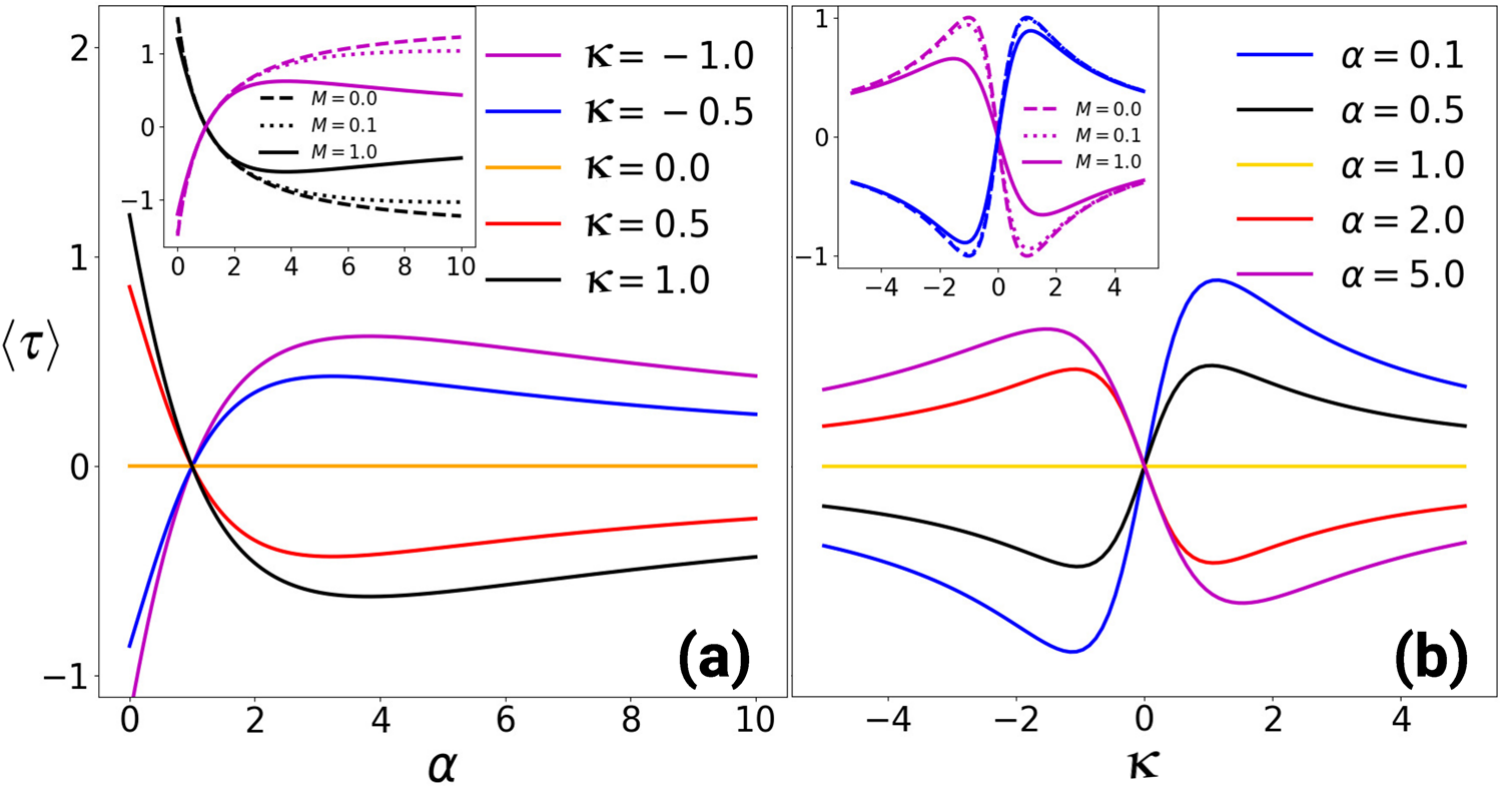
Average exerted torque by the magneto-gyrator as a function of (**a**) the parameter $\alpha$ for different values of $\kappa$ (**b**) diffusive Hall parameter $\kappa$ for different values of $\alpha$ with $T_h=4T_c=4.0$. The main figures show the results for $M=1.0$ while the insets depict those for $M=0.1$ and $M=0.01$ where $M=km/\gamma ^2$. (**a**) shows that the average torque has a maximum for an optimal value of $\alpha$ if the mass is sufficiently large. The maximum can be tuned by the applied magnetic field. As shown in (**b**), the average torque from Eq. (4) shows a maximum at an optimal $\kappa$, which can be reversed by the applied magnetic field. In addition, there is no torque if $\alpha =1$ or $\kappa = 0$, which corresponds to the stalled magneto-gyrator.

Our proposed Brownian magneto heat pump is made of a single Brownian particle with mass *m*, and charge *q* steered by a constant magnetic field *B*. The magnetic field is applied to the ${\hat{z}}$ direction such that ${\varvec{B}}= B{\hat{z}}$ and hence the particle’s motion along this direction is not affected by the applied magnetic field. Consequently, we effectively have a two-dimensional system with the particle’s position ${\varvec{r}}=(x, y)^\top$ where $\top$ indicates the transpose. The thermal fluctuations of unequal strength, proportional to the cold and hot heat bath temperatures $T_c$ and $T_h$, are supplied along the two Cartesian coordinates ${\hat{x}}$ and ${\hat{y}}$, respectively. By confining the particle via the potential $V({\varvec{r}})=\frac{1}{2}{\varvec{r}}^\top \cdot {\varvec{U}}\cdot {\varvec{r}}$, it performs an average gyrating motion. Here ${\varvec{U}}=k\mathop {}\!\varvec{\mathrm {diag}}(1, \alpha )$ is a diagonal matrix where *k* is the stiffness of the potential and $\alpha$ is a dimensionless parameter quantifying the difference in the stiffness in the *x* and *y* directions. Note that the essential condition for the particle to gyrate around the potential energy minimum is $\alpha \ne 1$. We model the dynamics of such a magneto-gyrator via the following underdamped Langevin equations:

1 $$\begin{aligned} m{\dot{v}}_x= & {} -\gamma v_x+\gamma \kappa v_y-kx + \xi _x(t) , \end{aligned}$$

2 $$\begin{aligned} m{\dot{v}}_y= & {} -\gamma v_y-\gamma \kappa v_x-k\alpha y + \xi _y(t) , \end{aligned}$$

where $v_x={\dot{x}}$ and $v_y={\dot{y}}$ are the velocities of the particle in *x* and *y* directions, respectively, $\gamma$ is the friction coefficient, and $\kappa =qB/\gamma$ is the diffusive Hall parameter which quantifies the strength of the Lorentz force relative to the frictional force. The stochastic force $\varvec{\xi }(t) =(\xi _x, \xi _y)^\top$ is Gaussian white noise with zero mean and time correlation $\langle \varvec{\xi }(t)\varvec{\xi }^{\top }(t')\rangle = 2\gamma \mathop {}\!\varvec{\mathrm {diag}}(T_c, T_h)\delta (t-t')$. Throughout this work we set the Boltzmann constant $k_B$ to unity.

To obtain a simple scalar quantifier for the strength of the gyrating current field, which emphasizes the tunability of the magneto-gyrator, we investigate the average torque on the potential. The average exerted torque by the particle on the potential *V*, denoted by $\langle \tau \rangle$, can be calculated as

3 $$\begin{aligned} \langle \tau \rangle = \int \rho ({\varvec{r}}) ({\varvec{r}}\times {\varvec{F}}_c) \mathop {}\!\mathrm {d}{\varvec{r}}, \end{aligned}$$

which is exactly equal to the opposite torque which the particle exerts via the friction forces on the thermal environment^53^. Here $\rho ({\varvec{r}})$ is the steady-state probability density of finding the particle at position ${\varvec{r}}$ and the conservative force is ${\varvec{F}}_c=-\nabla V({\varvec{r}})$. The average exerted torque on the potential by the particle becomes

4 $$\begin{aligned} \langle \tau \rangle = \frac{2\kappa (1-\alpha )(T_h-T_c)}{(\alpha -1)^2M+2(1+\alpha )(1+\kappa ^2)}. \end{aligned}$$

where $M=km/\gamma ^2$ is a dimensionless parameter.

Figure 2 shows how the average exerted torque by the magneto-gyrator varies by tuning (a) the stiffness of the potential for different values of the diffusive Hall parameter and (b) the diffusive hall parameter for different values of the parameter $\alpha$. The main figures represent the results for the magneto-gyrator with $M=1.0$ while those for smaller masses, corresponding to the overdamped dynamics, are shown in the insets. From Eq. (4) it is clear that the average torque is zero in the absence of a magnetic field or equivalently the magneto-gyrator is stalled, which is shown in the main figure in (a). In Fig. 2b, we show that there exists a maximum torque exerted by the magneto-gyrator at an optimal magnetic field. The average torque can be reversed by reversing the direction of the magnetic field.

### Brownian magneto-gyrator as a heat engine

**Figure 3:**
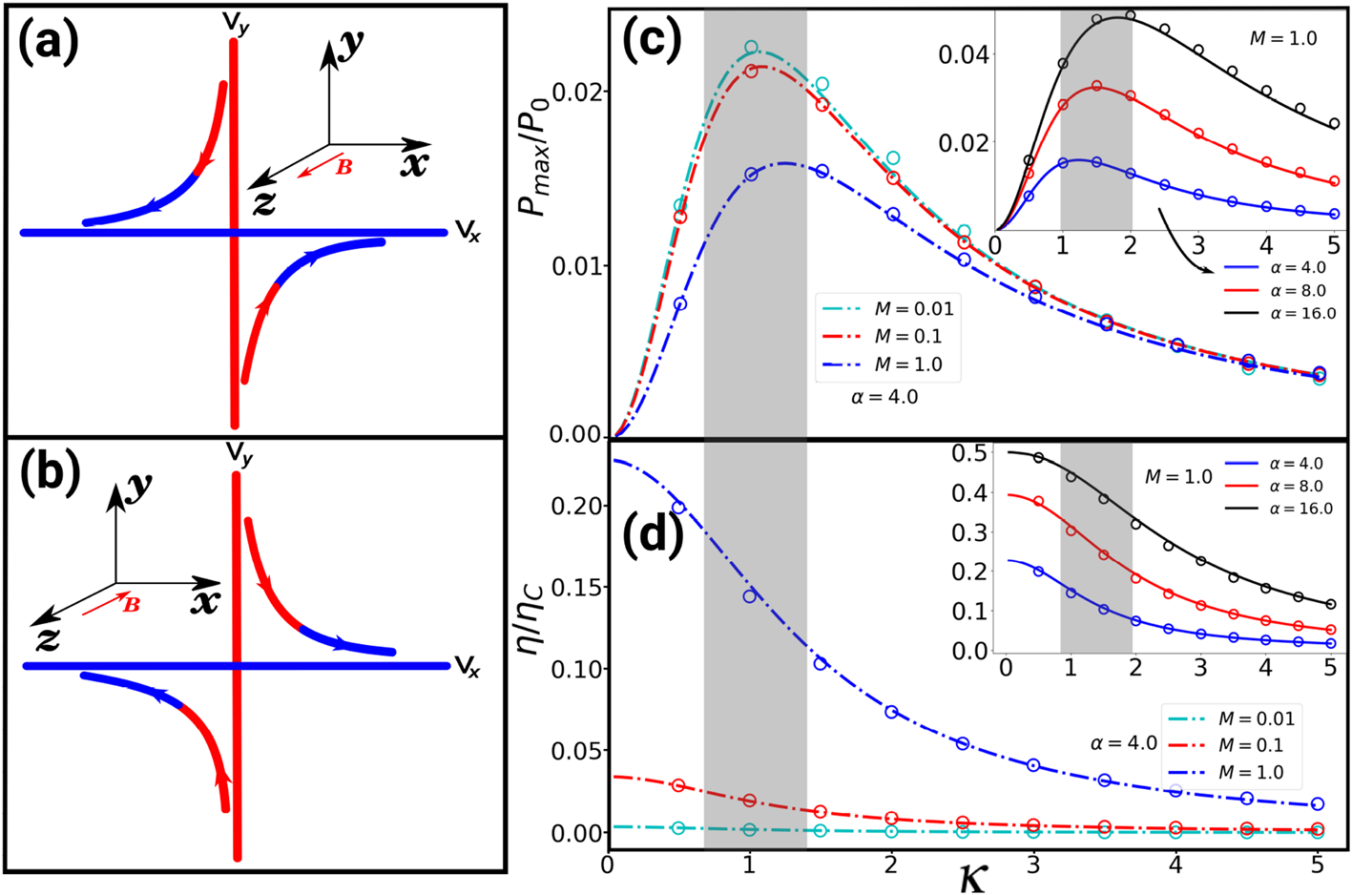
(**a**) and (**b**) show the schematics of the heat transfer in the velocity space due to an external magnetic field in the *z* and $-z$ directions, respectively. The velocity component which is coupled to the hot heat bath picks up higher kinetic energy, which due to the Lorentz force, gets transferred to the velocity component which is coupled to the cold heat bath on the time scale $m/\gamma$. This results in a heat transfer from the hot to the cold bath, shown in red and blue, respectively, in the velocity space. In (**c**), we represent the scaled maximum power with respect to the diffusive Hall parameter for $\alpha =4.0$, $T_h=4T_c=4.0$, and different values of *M* where $M=km/\gamma ^2$ and $P_0=(T_c + T_h)/\gamma$. In (**d**) we show the scaled efficiency $\eta /\eta _{c}$ corresponding to scaled maximum power in (**c**). While the maximum power decreases with increasing *M*, the corresponding efficiency increases due to the reduction in heat loss in the velocity space. The insets of (**c**) and (**d**) depict the scaled maximum power and the scaled efficiency for $M=1.0$ and different values of $\alpha$, respectively. The maximum power and corresponding efficiency increases with increasing $\alpha$. The shaded regions in (**c**) and (**d**) mark the $\kappa$ region in which the magneto-gyrator is operating close to maximum power and the corresponding efficiency. The lines show the theoretical predictions and the symbols are simulation results.

While performing gyration, a magneto-gyrator can be operated as a heat engine under a load. The engine dissipates heat at a steady rate while delivering output power. We first calculate the average mechanical power and then the corresponding heat loss to obtain the efficiency of the engine. We load the engine by applying a linear external nonconservative force ($\nabla \times {\varvec{F}}_{nc}\ne {\varvec{0}}$) of the form ${\varvec{F}}_{nc} = \varepsilon (-y, x)^\top$ with a parameter $\varepsilon >0$, yielding a torque in the *z* direction, whose sign is chosen such that the resulting torque in the *z* direction is opposed to $\langle \tau \rangle$ in Eq. (4). The goal is to calculate the average power of the work done by this force. The average extracted mechanical power $P=-\langle {\varvec{F}}_{nc}^\top .{\dot{{\varvec{r}}}}\rangle$ in the stationary state can be rewritten as $P=-\varepsilon \langle xv_y-yv_x\rangle$. Therefore, one needs to calculate the stationary-state position-velocity correlation matrix, that is $\lim _{t\rightarrow \infty }\langle {\varvec{r}}(t){\dot{{\varvec{r}}}}^\top (t)\rangle$. Note that by applying a prescribed external vortex flow field such as a rotating bucket to an underdamped Brownian particle one can induce similar terms to the nonconservative force^54^. Moreover, it is known that in experiments using optical tweezers, optical scattering forces actually generate a nonconservative component to the overall force exerted by the trap^55^. Here we take a linear nonconservative force for the convenience of analytical calculations.

The average mechanical power can be written as

5 $$\begin{aligned} \frac{P}{P_0} = \frac{4\frac{\varepsilon }{\varepsilon _s}\left( 1-\frac{\varepsilon }{\varepsilon _s}\right) }{G_1 - 4M(\frac{\varepsilon }{\varepsilon _s}+ G_2)^2}, \end{aligned}$$

where $P_0=k(T_c+T_h)/\gamma$, $G_1=k^2(1-\alpha )^2M/(\varepsilon _s^2(1+\kappa ^2))+k^2(2M(1+\alpha )+\kappa ^2)/(\varepsilon _s^2 M)$, and $G_2=k\kappa /(2\varepsilon _s M)$ (see Methods and Supplemental Materials). The mass-independent stall parameter $\varepsilon _s$ quantifies the maximum strength of the nonconservative force that one can apply before the engine stops working or equivalently the particle performs no gyration on average. Hence, the system operates as a heat engine, delivering mechanical work for $0< \frac{\varepsilon }{\varepsilon _s} < 1$. The stall parameter is given as

6 $$\begin{aligned} \varepsilon _s=k\frac{\kappa (\alpha -1)\eta _c}{2(2-\eta _c)(1+\kappa ^2)}, \end{aligned}$$

where $\eta _c=1-T_c/T_h$ is the Carnot efficiency.

In addition to the average mechanical power delivered by the engine, the average rate of heat out of the hot bath is needed to determine the efficiency $\eta$ of the engine. The absorbed heat by the particle from the cold and the hot baths, connected to the $i=x, y$ degrees of freedom,   and  , respectively, can be written as7where $\mathop {}\!\mathrm {d}W_i=\int _t^{t+\mathop {}\!\mathrm {d}t}\xi _i(t')\mathop {}\!\mathrm {d}t'$ with zero mean and $\langle \left( \mathop {}\!\mathrm {d}W_i\right) ^2\rangle =2\gamma T_i\mathop {}\!\mathrm {d}t$ and $\circ$ indicates the product in the Stratonovich sense.

Taking into account the nonconservative force ${\varvec{F}}_{nc}$ in Eq. (1) and Eq. (2), the heat in Eq. (7) can be rewritten as89The total derivatives in Eqs. (8) and  (9) have no contribution to the steady-state averages. The total heat flux (i.e. the sum of Eqs. (8) and  (9)) is the rate of work done by the engine whose average is given in Eq. (5). The average rate of the heat out of the hot bath,  and cold bath,  can be calculated which for the former reads as

10 $$\begin{aligned} Q_h = \gamma \kappa \langle v_xv_y\rangle - \frac{\varepsilon }{2} \langle xv_y-yv_x\rangle - \frac{\varepsilon }{2} \langle yv_x+xv_y\rangle , \end{aligned}$$

where . While the last term on the right hand side of Eq. (10) is zero (see the Supplemental Materials), the first term is the heat flow via momenta correlations whose origin can be understood as follows: in a magneto-gyrator the coordinate coupled to the hot thermostat picks up higher kinetic energy which, due to the Lorentz force, gets transferred to the coordinate coupled to cold thermostat on the time scale $m/\gamma$ (see Fig. 3a and b). This is analogous to the phonon Hall effect: in a paramagnetic dielectric subjected to a longitudinal temperature gradient, a magnetic field induces a transverse heat current perpendicular to the pre-existing temperature gradient^48^. The steady-state of a magneto-gyrator is thus characterised by correlations between different velocity components^56^.

Since the velocity correlation due to the magnetic field depends on the mass of the particle, the average heat out of the hot reservoir can be written as $Q_h=f(\kappa ,M,\varepsilon )/M$. The function *f* and the details are given in the Supplemental Materials. In the limit of zero mass, the function *f* remains finite giving rise to a divergent heat loss. In fact, in the overdamped approximation, the velocity correlation timescale is assumed to be infinitesimal. As in a Brownian magneto-gyrator heat flow between different reservoirs is mediated by the velocities, the overdamped approximation leads to a divergent heat flow between the reservoirs^44,47^. In contrast, in a Brownian gyrator the heat flux is governed by position-velocity correlations and hence there is no divergence of heat flux in the limit corresponding to zero mass^57^. Therefore, for our proposed engine, even in the overdamped regime the knowledge of particle’s mass is needed for the calculation of the heat flow and consequently the efficiency of the engine in converting the heat out of the hot bath into the power, $\eta =P/Q_h$, which is calculated in the Supplemental Materials. Note that the average torque and the average mechanical power in Eq. (4) and Eq. (5) remain well-defined in limit of *M* going to zero and therefore can be obtained in the overdamped approximation (see Supplemental Materials).

**Figure 4:**
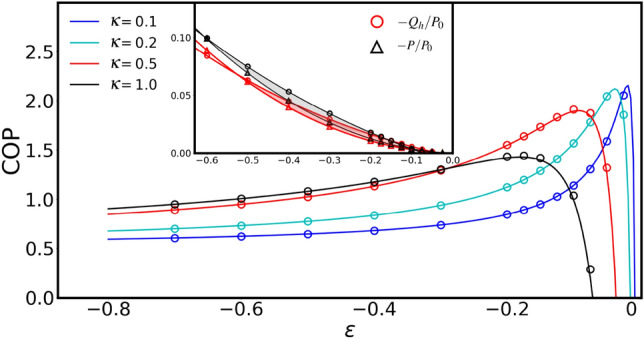
The main figure shows the coefficient of performance of a Brownian magneto-refrigerator with $T_h/T_c=1.1$, and $M=1.0$, $\alpha =4.0$ as a function of the force strength $\varepsilon$ for different values of the diffusive Hall parameter $\kappa$. The sign of the force strength $\varepsilon$ is such that the resulting torque in the *z* direction is along the same direction as the torque due to the applied magnetic field. The COP of the refrigerator has a maximum for each value of $\kappa$. The inset shows the average rate of the heat absorbed by the hot bath and the corresponding input power where $P_0=k(T_c+T_h)/\gamma$. The red and gray shaded regions correspond to a refrigerator with $\text {COP}>1$ for $\kappa =0.5$ and $\kappa =1.0$, respectively. The solid lines are from our theoretical predictions and the symbols depict the simulation results.

Carnot efficiency can be obtained for an infinitely slow transformation, which for the magneto-gyrator corresponds to a stalled engine with large *M*. Due to this, a more useful notion is that of efficiency at maximum power^31^. In Fig. 3c and d we show the scaled maximum power, $P_{max}/P_0$ and the corresponding scaled efficiency, $\eta /\eta _c$ in terms of the parameter $\kappa$ for $\alpha =4.0$, $T_h=4T_c=4.0$, and different values of *M*, respectively. While the maximum power decreases with increasing *M*, the corresponding efficiency increases due to the reduction in heat loss in the velocity space. In insets of (c) and (d) show the scaled maximum power and the scaled efficiency for $M=1.0$ and different values of $\alpha$, respectively. One could operate the magneto-gyrator as following: For a given *M* and $\alpha$, tune the magnetic field so that the magneto-gyrator operates at the maximum power as shown in the shaded region in Fig. 3c with a corresponding efficiency at maximum power in Fig. 3d. Note that the dimensionless mass $M=1.0$ is achievable for a particle with a radius $R\sim 10^{-5}m$ and mass $m\sim 10^{-11} kg$ (such as spherical PMMA particles) in a potential with an optical trapping stiffness $k\sim 10^{-6}N/m$, as used in Ref.^29^, in a solvent of a viscosity $\nu \sim 10^{-5} N.s/m^2$ such as a dusty plasma. This implies that for the parameters $\alpha = 4.0$ and $M = 1.0$, magneto-gyrator will deliver maximum possible power at $\kappa \approx 1$ with an efficiency $\approx 0.15\eta _{c}$ for an experimentally realisable temperature ratio $T_h/T_c = 4.0$^29^. However, as we show in the inset, the efficiency at maximum power can approach $0.5\eta _c$ for $\alpha =16.0$. We note that the stationary-state averages are meaningful only for a stable magneto-gyrator. In the Supplemental Materials we find the generalised stability condition as $G_1 - 4M(\frac{\varepsilon }{\varepsilon _s}+ G_2)^2>0$ which reduces to known results in Ref.^58^ for $\alpha =1.0$. We show that our engine is stable for the chosen parameters in this work.

### Brownian magneto-refrigerator

Onsager symmetry implies that a Brownian heat pump can be operated as a refrigerator^9–11^. In the context of refrigeration, the driving mechanism is based on the rectification of thermal fluctuations^16,59^. Usually, this requires the engine to be loaded beyond stalling. In the context of a Brownian gyrator, which also serves as a heat pump, we have analytically and numerically checked that this corresponds to either loading until the gyration direction reverses due to the external work on the engine or supporting the gyration of the engine (see Fig. 1b). Under this protocol, heat can be extracted from the cold bath to perform cooling, i.e, refrigeration. Similarly, heat can be dumped into the hot bath. We use the following protocol to operate a magneto-gyrator as a refrigerator: performance of work on the system by enhancing the gyration along the direction of the magneto-gyrator. Our analysis is not limited to the linear response regime and provides a full response. We note that a magneto-gyrator under loading can not perform cooling; the absorbed heat from the hot bath increases under loading monotonically.

To perform work on the system, we consider the linear nonconservative force ${\varvec{F}}_{nc} = \varepsilon (-y, x)^\top$ with a negative force strength $\varepsilon < 0$ such that the resulting torque in the *z* direction is along the same direction as the average torque due to the magnetic field, given in Eq. (4). We operate the refrigerator in a range of $\varepsilon$ where the system is stable. By doing so, the input power of the Brownian magneto-refrigerator is given as $P=-\varepsilon \langle xv_y-yv_x\rangle$. The average rate of the absorbed heat from the hot bath, $Q_h$, is given in Eq. (10). The coefficient of performance (COP) of the refrigerator can be measured as $Q_h/P$, which is given in the Supplemental Materials. In this work, we investigate the COP of the hot bath, however the COP of the cold bath can be equivalently obtained as $Q_c/P$. Note that the sum of the COPs of the cold and hot baths is unity.

**Figure 5:**
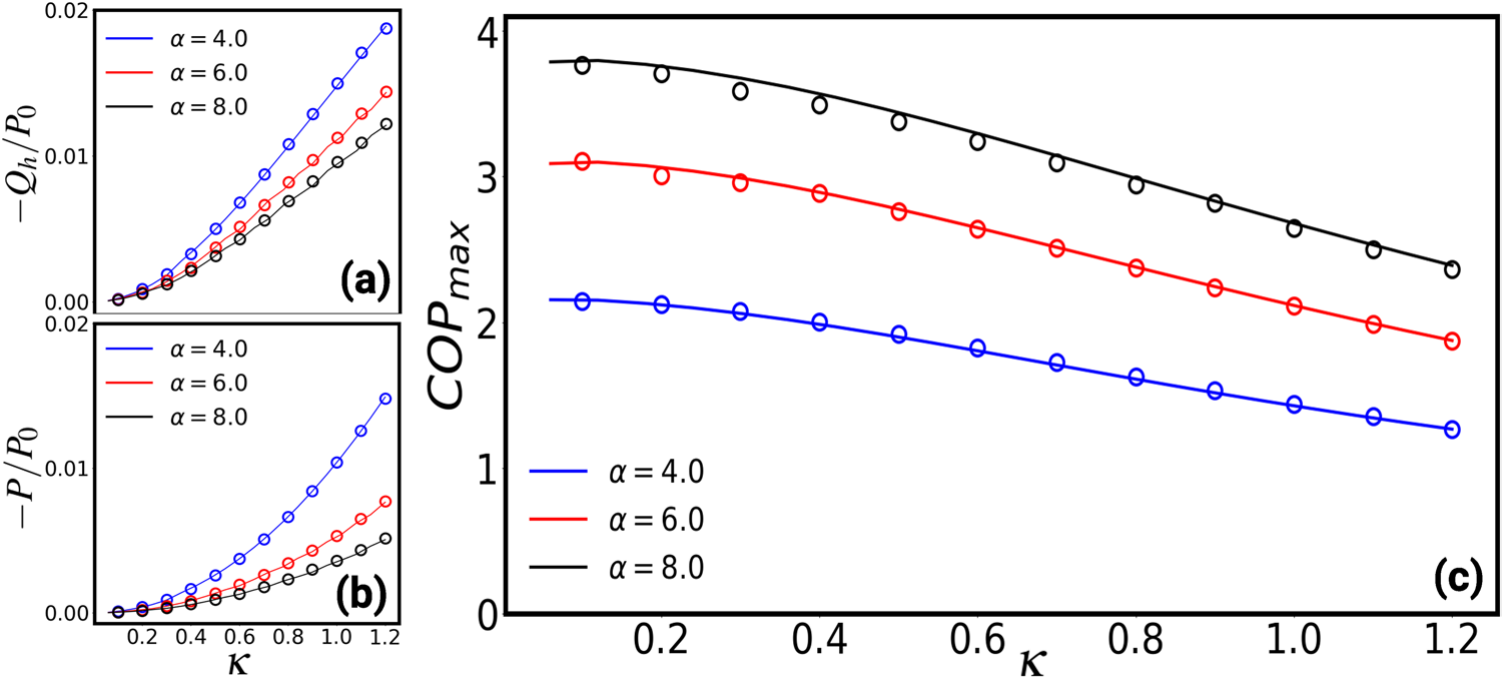
(**a**) and (**b**) present the average rate of the heat absorbed by the hot bath and the corresponding input work for a magneto-refrigerator operating at maximum COP with $T_h/T_c=1.1$ and $M=1.0$ as a function of the diffusive Hall parameter parameter $\kappa$ for different values of $\alpha$ where $P_0=k(T_c + T_h)/\gamma$. Note that the work done on the system is negative giving rise to a negative heat flux. (**c**) shows the maximum of the COP of the corresponding system. The solid lines depict the theoretical predictions and the symbols show the simulation results. The maximum COP is larger than unity for a range of $\kappa$ shown in this figure.

Figure 4 shows the COP of a Brownian magneto-refrigerator with respect to the force strength $\varepsilon$. The work done on the system is negative giving rise to a negative heat flux. The COP of the magneto-refrigerator can be tuned and optimised by the applied magnetic field. As can be seen in Fig. 4, the COP of the refrigerator is larger than unity for $\kappa \lessapprox 1$. In the inset, we show the average rate of the heat absorbed by the hot bath and the corresponding input power. The red and gray shaded regions show where the refrigerator can be operated with $\text {COP}>1$ for $\kappa =0.5$. The COP of the refrigerator maximizes at an optimal $\varepsilon$ for each value of the diffusive Hall parameter $\kappa$. Figure 5 (a) and (b) present the average rate of the heat absorbed by the hot bath and the corresponding input work for a magneto-refrigerator operating at maximum COP. In Fig. 5 we show the maximum COP with respect to the diffusive Hall parameter. For the same parameters as proposed above for a realisable heat engine and with the temperature ratio $T_h/T_c=1.1$, a magneto-refrigerator with $\text {COP}>2$ can be realised.

## Discussion

In this paper, we propose a Brownian magneto heat pump steered by an external magnetic field which provides a novel way of tuning the properties of the pump. We show that while performing gyration such a magneto-gyrator can be operated as both a heat engine and a refrigerator whose performance can be tuned and optimised by the applied magnetic field. The field also gives the tunability of the key properties of the engine, such as the direction of gyration, the torque, and the output power and those of the magneto-refrigerator, like the average rate extracted heat from the cold bath or similarly the average rate of the heat dumped into the hot bath and the corresponding input work. Further tunability can be achieved by combining the ideas between a Brownian gyrator and a magneto gyrator, as we elaborate in the Supplemental Materials.

The working principle of the proposed magneto-gyrator is the stationary-state correlations between the spatial degrees of freedom arising from the velocity correlations. These correlations are induced by the Lorentz force and do not require special alignment of the confining potential with respect to the temperature axes, as in the previous studies^47,53,60–66^. In fact, Lorentz force-induced correlations exist even for a freely diffusing Brownian particle subjected to two different thermostats^56^. The correlations, however, vanish for large magnetic fields; the diffusion becomes smaller with increasing magnetic field. This implies that there exists an optimal magnetic field to operate the magneto-gyrator as a heat engine, as reflected by the existence of a maximum in the output power as a function of the diffusive Hall parameter.

The momenta correlations in a magneto-gyrator give rise to heat flow, which is analogous to the phonon Hall effect. As in the phonon Hall effect, the rate of heat flow can be tuned by the applied magnetic field. Our proposed magneto-gyrator could be a possible realisable system to measure the momenta governed heat transfer induced by the magnetic field.

Finally, we consider the possibility of an experimental realisation of the proposed magneto heat pump. A possible experimental set up is to trap the particle using optical tweezers subjected to a fluctuating electric field either in a radio-frequency plasma sheath with a vertical magnetic field^67,68^ or in a rotating frame of reference. In the latter case, a well-controlled rotation of the reference frame induces a Coriolis force which acts the same as the Lorentz force due to an external magnetic field^52,69,70^. Based on these experimental studies, it seems that a magneto-refrigerator could be realised in dusty plasmas with $\text {COP}>2$ at experimentally realisable magnetic fields. Similarly, a heat engine with an efficiency of $\sim 0.15 \eta _{c}$ could be realised while delivering maximum power. Another possible realisation could be an experiment with laser cooled atomic system in which one can create different temperatures along the different cooling axis via laser detuning imbalance during the cooling phase in which mean rotation of atoms can be observed^61^. Our initial finding of refrigeration motivates us to make the following Gedankenexperiment: Consider two different magneto-gyrators, each with its own set of thermostats and confining potentials. If one couples the two Brownian particles, for instance, via a Hookean spring, and subject the two gyrators to different magnetic fields, we have effectively one gyrator acting as an engine which can perform work on the other gyrator which may function as a refrigerator. Such coupled gyrators do not require an external protocol for loading, for instance, the non-conservative rotating force considered in our work and others. Such a set-up could be a promising experimental realisation of coupled gyrators.

From a future perspective, we plan to go beyond the description of the magneto heat pump in terms of its average properties. We plan to use stochastic thermodynamics to calculate the fluctuations in torque and work delivered by the magneto-gyrator. Furthermore, it could be interesting to investigate a possible mass separation in a system of various masses: the stability condition of the magneto-gyrator implies that the larger the mass of the particle is the earlier the particle escapes from the trap. Our findings might be relevant to skyrmions^71^. It has been recently shown that when subjected to radial thermal gradients, skyrmions spontaneously start rotating giving rise to a ratchet motion^72^. It would be interesting to investigate whether trapped skyrmions subjected to two different thermostats, as in our magneto-gyrator, exhibit spontaneous chirality which could be used to extract work. Finally, our analysis might be applicable to other systems which exhibit circular motion in an anisotropic fluctuation field including chiral colloidal microswimmers in parabolic potentials^73^, active Janus particles in a complex plasma^74^, particles dominated by the Magnus force^75^.

## Methods

### Steady-state solution

In this section, we present the method that we used to calculate stationary-state covariance matrix whose elements are given in the Supplemental Materials. The two-dimensional motion of a charged, Brownian particle of mass *m*, trapped in an external potential $V({\varvec{r}})$, in the presence of an external magnetic field *B* in the *z* direction and the nonconservative force ${\varvec{F}}_{nc}=\varepsilon (-y, x)$ can be described by the following underdamped Langevin equation

11 $$\begin{aligned} {\dot{{\varvec{z}}}}(t) = -{\varvec{F}}{\varvec{z}}(t) + {\tilde{\varvec{\xi }}}(t), \end{aligned}$$

where ${\varvec{z}}(t)=(x(t), y(t), v_x(t), v_y(t))^\top$ . Here  ${\tilde{\varvec{\xi }}}(t)=(0, 0, m^{-1}\xi _x(t), m^{-1}\xi _y(t))^\top$ is Gaussian white noise with zero mean and Dirac delta time correlation $\langle {\tilde{\varvec{\xi }}}(t){\tilde{\varvec{\xi }}}^{\top }(t')\rangle = (2\gamma /m^2)\varvec{\mathrm {T}}\delta (t-t')$ where $\gamma$ is the constant friction coefficient. Here *k* is the stiffness of the potential, $\alpha$ is a dimensionless parameter, and $\varvec{\mathrm {T}}=\mathop {}\!\varvec{\mathrm {diag}}(0, 0, T_c, T_h)$ is a diagonal matrix. The matrix ${\varvec{F}}$ is defined as

12 $$\begin{aligned} {\varvec{F}}= \frac{1}{m}\left( \begin{array}{cc} {\varvec{0}} &{} -m{\mathbb {I}} \\ {\varvec{U}}_l &{} {\varvec{G}}\\ \end{array}\right) , \end{aligned}$$

where ${\mathbb {I}}$ is the identity matrix and

13 $$\begin{aligned} {\varvec{G}}= \gamma \left( \begin{array}{cc} 1 &{} -\kappa \\ \kappa &{} 1 \\ \end{array}\right) , {\varvec{U}}_l = k\left( \begin{array}{cc} 1 &{} \varepsilon ^\prime \\ -\varepsilon ^\prime &{} \alpha \\ \end{array}\right) . \end{aligned}$$

where $\varepsilon ^\prime =\varepsilon /k$ is a dimensionless parameter. In order to calculate the COP of a magneto-refrigerator and the efficiency of a magneto heat engine the calculation of the rate of the heat flow, $\langle {\dot{Q}}_i\rangle$ and the average mechanical power, *P* is needed, which can be determined by the steady-state covariance matrix $\varvec{\mathrm {S}}=\lim _{t\rightarrow \infty }\varvec{\mathrm {S}}(t)$, where $\varvec{\mathrm {S}}(t)=\langle {\varvec{z}}(t){\varvec{z}}^\top (t)\rangle$. The change in the covariance matrix in the time interval $\mathop {}\!\mathrm {d}t$ is given by

14 $$\begin{aligned} \mathop {}\!\mathrm {d}\varvec{\mathrm {S}}(t)=-[{\varvec{F}}\varvec{\mathrm {S}}(t)+\varvec{\mathrm {S}}(t){\varvec{F}}^\top ]\mathop {}\!\mathrm {d}t +\int _t^{t+\mathop {}\!\mathrm {d}t}\mathop {}\!\mathrm {d}t^\prime \int _t^{t+\mathop {}\!\mathrm {d}t}\mathop {}\!\mathrm {d}t^{\prime \prime }\langle {\tilde{\varvec{\xi }}}(t'){\tilde{\varvec{\xi }}}^\top (t^{\prime \prime })\rangle , \end{aligned}$$

which using the property of the noise ${\tilde{\varvec{\xi }}}$, the time evolution of the covariance matrix can be written as

15 $$\begin{aligned} \frac{\mathop {}\!\mathrm {d}\varvec{\mathrm {S}}(t)}{\mathop {}\!\mathrm {d}t}=-{\varvec{F}}\varvec{\mathrm {S}}(t)-\varvec{\mathrm {S}}(t){\varvec{F}}^\top +\frac{2\gamma }{m^2}\varvec{\mathrm {T}}. \end{aligned}$$

The stationary-state covariance matrix can be calculated by setting $\mathop {}\!\mathrm {d}\varvec{\mathrm {S}}(t)/\mathop {}\!\mathrm {d}t$ to zero, which consists of the steady-state position-position, position- and velocity-velocity correlations. The solution to Eq. (15) gives the stationary-state covarinace matrix with the elements which are given in the Supplemental Materials.

### Brownian dynamics simulations

To validate our theoretical predictions and confirm the stability of the system we perform Brownian dynamics simulations using the Langevin equations of motion. We use the underdamped Langevin equations, given in Eqs. (1) and (2), with $M=1.0$, $M=0.1$, and $M=0.01$ the integration time steps $dt=1 \times 10^{-3}\tau$, $1 \times 10^{-4}\tau$, and $1 \times 10^{-6}\tau$, respectively, where $\tau =\gamma /k$ serves as a natural time scale which we set to unity.

## Supplementary Information

Supplementary Information.

## Data Availability

The data that support the findings of this study are available from the authors upon reasonable request.

## Code availability

The code that supports the findings of this study are available from the authors upon reasonable request.
