# Supplementary material for: Tunable Brownian magneto heat pump
Source: Sci Rep. 2022 Aug 4;12:13405. doi: 10.1038/s41598-022-17584-3 (PMC9352690; doi:10.1038/s41598-022-17584-3)
Supplement: Supplementary file 1 — Supplementary Information. [file 41598_2022_17584_MOESM1_ESM.pdf]

Supplemental Materials: Tunable Brownian magneto heat pump

Iman Abdoli[1], René Wittmann[2], Joseph Michael Brader[3], Jens-Uwe Sommer[1,4], Hartmut Löwen[2], and Abhinav Sharma[1,4,*]

[1]Leibniz-Institut für Polymerforschung Dresden, Institut Theorie der Polymere, Dresden, 01069, Germany
[2]Institut für Theoretische Physik II, Weiche Materie, Heinrich-Heine-Universität Düsseldorf, Düsseldorf, 40225, Germany
[3]Department de Physique, Université de Fribourg, CH-1700 Fribourg, Switzerland
[4]Technische Universität Dresden, Institut für Theoretische Physik, Dresden, 01069, Germany
[*]sharma@ipfdd.de

ABSTRACT

In the Supplemental Materials, we first investigate the overdamped approximation of a Brownian magneto-gyrator: We take the low-mass approach to derive the proper overdamped Langevin equation describing the dynamics of such a microengine. We calculate the stationary-state quantities of the microengine, such as the average exerted torque by the microengine on the confining potential and mechanical power, and study the tunability of the engine via the applied magnetic field. Next, we obtain the exact expressions for the efficiency of a magneto heat engine and the COP of a magneto-refrigerator in the underdamped regime, as discussed in the main text.

Low-mass Limit of Langevin Equation

In the presence of a magnetic field, the overdamped Langevin equation can not be derived by setting the inertial term to zero. Anomalous fluxes has been observed in overdamped Brownian dynamics by setting the inertial term to zero in the velocity Langevin equation due to Lorentz force[1]. Here we take the low-mass approach[2] to derive the overdamped Langevin equation describing the dynamics of a charged Brownian particle with mass m and charge q subjected to a magnetic field B in the $\hat{z}$ direction. The velocity Langevin equation for the position $\boldsymbol{r} = (x,y)^\top$ and the velocity $\boldsymbol{v} = (v_x, v_y)$ of the particle under the effect of the external force $\boldsymbol{f}(\boldsymbol{r}) = (f_x(\boldsymbol{r}), f_y(\boldsymbol{r}))^\top$, can be written as

$$m\dot{\boldsymbol{v}} = \boldsymbol{f}(\boldsymbol{r}(t)) - \boldsymbol{G}\boldsymbol{v}(t) + \boldsymbol{\xi}(t), \tag{1}$$

where $\boldsymbol{\xi}(t) = (\xi_x(t), \xi_y(t))^\top$ is the Gaussian white noise with zero mean and Dirac delta correlation $\langle \boldsymbol{\xi}(t)\boldsymbol{\xi}^\top(t')\rangle = 2\gamma \mathrm{T}\delta(t-t')$ with γ being the friction coefficient and $\mathrm{T} = \boldsymbol{diag}(T_c, T_h)$. The matrix $\boldsymbol{G}$ is defined as

$$\boldsymbol{G} = \gamma \begin{pmatrix} 1 & -\kappa \\ \kappa & 1 \end{pmatrix}, \tag{2}$$

with $\kappa = qB/\gamma$ being the diffusive Hall parameter which quantifies the strength of the Lorentz force relative to the frictional force. By omitting the transient term $e^{-\boldsymbol{G}t/m}\boldsymbol{v}(0)$, which is negligible in the low-mass limit, the formal solution to Eq. (1) can be written as

$$\boldsymbol{v}(t) = \frac{1}{m}\int_0^t \mathrm{d}t' e^{-\frac{\boldsymbol{G}}{m}(t-t')}[\boldsymbol{f}(\boldsymbol{r}(t')) + \boldsymbol{\xi}(t')]. \tag{3}$$

which yields the following stochastic integrodifferntial equation

$$\dot{\boldsymbol{r}}(t) = \frac{1}{m}\int_0^t \mathrm{d}t' e^{-\frac{\boldsymbol{G}}{m}(t-t')}\boldsymbol{f}(\boldsymbol{r}(t')) + \boldsymbol{\chi}_m(t), \tag{4}$$

where $\boldsymbol{\chi}_m(t)$ is Gaussian non-white noise given as

$$\boldsymbol{\chi}_m(t) = \frac{1}{m}\int_0^t \mathrm{d}t' e^{-\frac{\boldsymbol{G}}{m}(t-t')}\boldsymbol{\xi}(t'). \tag{5}$$

The main purpose of this section is to derive the statistical properties of this noise and thereby the overdamped Langevin equation. From Eq. (5) it is clear that the first moment of the noise is zero, i.e., $\langle \boldsymbol{\chi}_m(t)\rangle = 0$. The interesting property of the noise is encoded in its time correlation $\langle \boldsymbol{\chi}_m(t)\boldsymbol{\chi}_m^\top(s)\rangle = \boldsymbol{\Sigma}_m(t,s)$ in the low-mass limit. Here $\boldsymbol{\Sigma}_m(t,s)$ is the correlation matrix and the subscript indicates the mass dependence of the quantity. The correlation matrix can be written as

$$\boldsymbol{\Sigma}_m(t,s) = \frac{1}{m^2}\int_0^s \mathrm{d}u \int_0^t \mathrm{d}t' e^{-\frac{\boldsymbol{G}}{m}(t-t')}\langle \boldsymbol{\xi}(t')\boldsymbol{\xi}^\top(u)\rangle e^{-\frac{\boldsymbol{G}^\top}{m}(s-u)}, \tag{6}$$

where using the properties of the Gaussian white noise $\boldsymbol{\xi}(t)$, in the limit that $t\to\infty$ and $s\to\infty$ and for a finite $t-s$, it can be rewritten as

$$\boldsymbol{\Sigma}_m(t,s) = \frac{2\gamma}{m^2} e^{-\frac{\boldsymbol{G}}{m}(t-s)} \lim_{s\to\infty}\int_0^s \mathrm{d}u \left[e^{-\frac{\boldsymbol{G}}{m}(s-u)}\mathrm{T}e^{-\frac{\boldsymbol{G}^\top}{m}(s-u)}\right], \tag{7}$$

if $t \geq s$ and

$$\boldsymbol{\Sigma}_m(t,s) = \frac{2\gamma}{m^2}\left[\lim_{t\to\infty}\int_0^t \mathrm{d}t' e^{-\frac{\boldsymbol{G}}{m}(t-t')}\mathrm{T}e^{\frac{-\boldsymbol{G}^\top}{m}(t-t')}\right] e^{-\frac{\boldsymbol{G}^\top}{m}(s-t)}, \tag{8}$$

if $t < s$. The integrals in Eq. (7) and Eq. (8) can be easily calculated, which yield

$$\boldsymbol{\Sigma}_m(t,s) = \begin{cases} \frac{1}{m}e^{-\frac{\boldsymbol{G}}{m}(t-s)}\boldsymbol{C}, & t\geq s,\\ \frac{1}{m}\boldsymbol{C}e^{-\frac{\boldsymbol{G}^\top}{m}(s-t)}, & t<s. \end{cases} \tag{9}$$

where the matrix $\boldsymbol{C}$ is defined as

$$\boldsymbol{C} = \frac{1}{2(1+\kappa^2)}\begin{pmatrix} 2T_c+\kappa^2(T_c+T_h) & \kappa(T_h-T_c)\\ \kappa(T_h-T_c) & 2T_h+\kappa^2(T_c+T_h)\end{pmatrix}. \tag{10}$$

Note that the cross-correlation components of the correlation matrix in Eq. (10) have their origin in the broken time-reversal symmetry of the magnetic field[1,2].

The correlation matrix $\boldsymbol{\Sigma}_m$ depends on $s' \equiv t-s$ and satisfies $\boldsymbol{\Sigma}_m(-s') = \boldsymbol{\Sigma}_m^\top(s')$. The elements of the correlation functions are given by

$$\boldsymbol{\Sigma}_m(s') = \frac{1}{m}\boldsymbol{C}^{1-a}e^{-\frac{\gamma}{m}|s'|}\begin{pmatrix} \cos\left(\frac{qB}{m}s'\right) & \sin\left(\frac{qB}{m}s'\right)\\ -\sin\left(\frac{qB}{m}s'\right) & \cos\left(\frac{qB}{m}s'\right)\end{pmatrix}\boldsymbol{C}^a. \tag{11}$$

where $a=0$ if $s'<0$ and $a=1$ otherwise. As m decreases, the correlation functions become singular with diverging oscillation frequency qB/m, diverging amplitude proportional to $1/m$, and vanishing decay time m/γ. It can be shown that for any function $h(s')$ with no singularity around $s'=0$, the low-mass limit yields

$$\lim_{m\to 0}\int_0^\infty \mathrm{d}s'\, h(s')\boldsymbol{\Sigma}_m(s') = h(0)\boldsymbol{G}^{-1}\boldsymbol{C}, \tag{12}$$

$$\lim_{m\to 0}\int_{-\infty}^0 \mathrm{d}s'\, h(s')\boldsymbol{\Sigma}_m(s') = h(0)\boldsymbol{C}(\boldsymbol{G}^{-1})^\top. \tag{13}$$

Therefore, the correlation matrix in the low-mass limit, i.e., $m\to 0$ can be written as

$$\boldsymbol{\Sigma}(s') \equiv \lim_{m\to 0}\boldsymbol{\Sigma}_m(s') = \boldsymbol{G}^{-1}\boldsymbol{C}\delta_+(s') + \boldsymbol{C}(\boldsymbol{G}^{-1})^\top\delta_-(s'), \tag{14}$$

where the notations $\delta_\pm(s')$ indicate the different Dirac delta functions which are zero for $s'\neq 0$ while $\int_0^\infty \mathrm{d}s'\delta_+(s') = \int_{-\infty}^0 \mathrm{d}s'\delta_-(s') = 1$ and $\int_0^\infty \mathrm{d}s'\delta_-(s') = \int_{-\infty}^0 \mathrm{d}s'\delta_+(s') = 0$.

To complete the derivation of the overdamped Langevin equation we now evaluate the first term on the right hand side of Eq. (4). Using the change of variable from t' to $s'=t-t'$ it can be written as

$$\frac{1}{m}\int_0^t \mathrm{d}t' e^{-\frac{\boldsymbol{G}}{m}(t-t')}\boldsymbol{f}(\boldsymbol{r}(t')) = \left[\int_0^t \mathrm{d}s'\ \boldsymbol{\Sigma}_m(s')\boldsymbol{f}(\boldsymbol{r}(t-s'))\right]\boldsymbol{C}^{-1}, \tag{15}$$

where using Eq. (12), in the low-mass limit converges to $\boldsymbol{G}^{-1}\boldsymbol{f}(\boldsymbol{r}(t))$. Thus the Langevin equation in the low-mass limit corresponding to the overdamped dynamics can be written as

$$\dot{\boldsymbol{r}}(t) = \boldsymbol{G}^{-1}\boldsymbol{f}(\boldsymbol{r}(t)) + \boldsymbol{\chi}(t), \tag{16}$$

where $\boldsymbol{\chi}(t)$ is Gaussian non-white noise with zero mean and the time correlation as in Eq. (14).

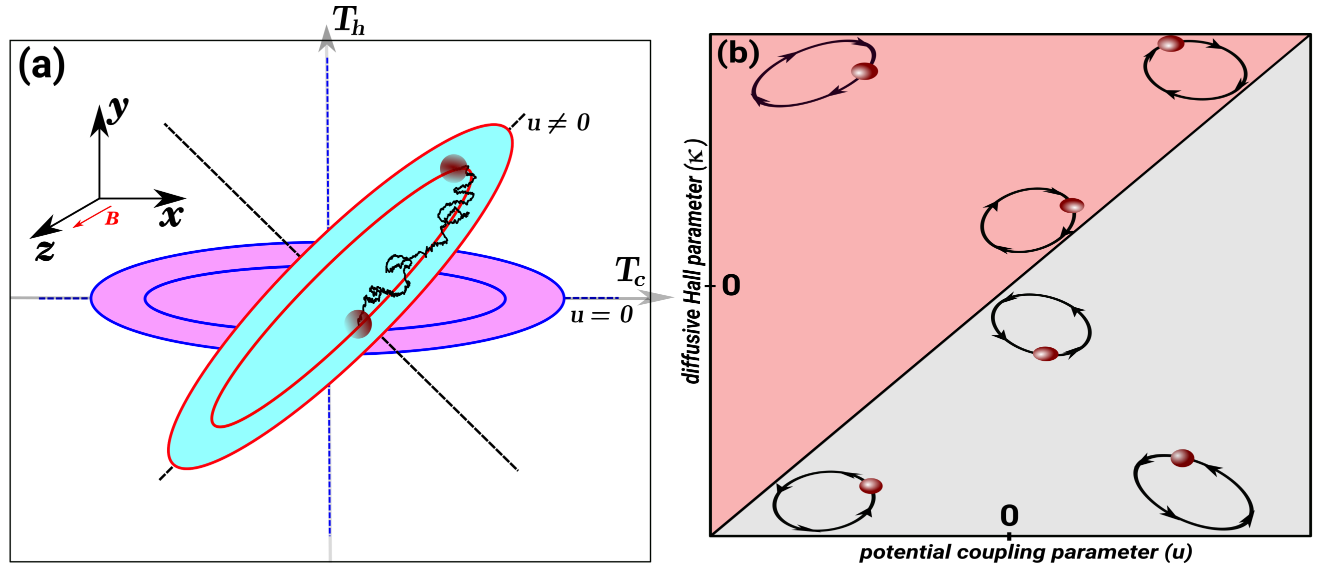

Figure 1. A single charged Brownian particle, steered by an external magnetic field B and trapped in a rotationally asymmetric potential, performs gyration when subjected to different thermal noises from cold, T_c, and hot, T_h, heat baths coupled to its x and y degrees of freedom, respectively. Figure (a) schematically depicts the diffusion of the particle in a magnetic field in the z direction and under the influence of the harmonic potential $V(x,y) = k[(x^2+\alpha y^2)/2 + uxy]$ with the parameters k and α. Here u is the potential coupling parameter which correlates the spatial degrees of freedom. The generic scenario is considered when the principal axes of the potential, shown by dashed lines, are misaligned with the temperature axes, namely if $u \neq 0$. Note that this condition (i.e., $u \neq 0$) is not necessary for the system to operate as an engine, yet is considered for further tunability of the microengine. (b) Schematic illustration of the average torque generated by the microengine for different values of the diffusive Hall parameter $\kappa = qB/\gamma$ and the potential coupling parameter u. The solid, oblique line represents zero torque in the system corresponding to stalled engine. The reddish region corresponds to the clockwise torque while the gray region depicts the counterclockwise torque. As can be seen, a torque can be generated even if the potential coupling parameter is zero upon applying an external magnetic field. The generated torque can be tuned by varying the magnitude and direction of the applied magnetic field: it changes the direction by reversing the direction of the applied magnetic field if $u = 0$ or by additionally changing the sign of the potential coupling parameter when it is not zero. Note that latter can be also done by further tunability of the applied magnetic field for a fixed u.

Brownian Magneto-Gyrator as a Tunable Microengine

A Brownian magneto-gyrator is made of a single Brownian particle with charge q steered by a constant magnetic field B and trapped by the conservative force $\boldsymbol{F}_c = -\nabla V(\boldsymbol{r})$. We consider a magnetic field in the $\hat{z}$ direction such that $\boldsymbol{B} = B\hat{z}$ and hence the particle's motion along this direction is not affected by the applied magnetic field. Consequently, we effectively have a two-dimensional system with the particle's position $\boldsymbol{r} = (x,y)^\top$ where $\top$ indicates the transpose. The thermal fluctuations of unequal strength, proportional to the cold and hot heat bath temperatures T_c and T_h, are supplied along the two Cartesian coordinates $\hat{x}$ and $\hat{y}$, respectively. Here $V(\boldsymbol{r}) = \frac{1}{2}\boldsymbol{r}^\top \cdot \mathrm{U} \cdot \boldsymbol{r}$ is the potential where the matrix U is given as

$$\mathrm{U} = k\begin{pmatrix} 1 & u \\ u & \alpha \end{pmatrix}, \tag{17}$$

where k is the stiffness of the potential, α is a dimensionless parameter quantifying the difference in the stiffness in the x and y directions, and u is the potential coupling parameter which correlates the spatial degrees of freedom. Note that the stability condition implies that $|u| < \sqrt{\alpha}$. The eigenvectors of the matrix U correspond to the principal axes of the potential, which are shown by dashed lines in Fig. 1 (a). This figure shows a schematic of the system where the alignment of the principal axes of the potential with the temperature axes is specified by the potential coupling parameter. In the case of $u = 0$ the principal axes of the potential align with the temperature axes. The overdamped dynamics of the system can be described by the following Langevin equation

$$\dot{\boldsymbol{r}}(t) = -\boldsymbol{A}\boldsymbol{r}(t) + \boldsymbol{\chi}(t), \tag{18}$$

where $\boldsymbol{A} = \boldsymbol{G}^{-1}\mathrm{U}$ and $\boldsymbol{\chi}(t)$ is Gaussian nonwhite noise[2] with zero mean and time correlation as in Eq. (14).

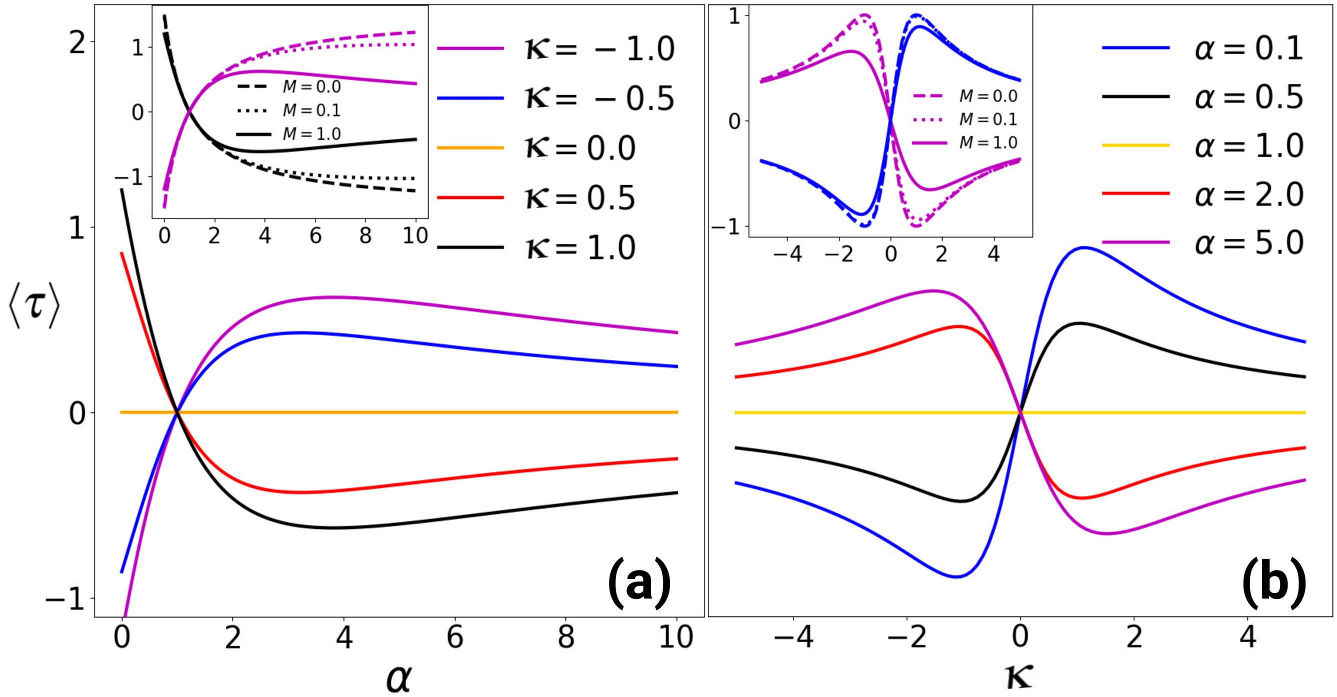

Figure 2. The left figure shows a panel of the stationary-state probability density of the particle's position and fluxes in the system for different values of the diffusive Hall parameter κ and the potential coupling parameter u. The results are shown from Eq. (22) in the stationary state for the probability density and substituting that equation into Eq. (20) for the fluxes with $T_h = 4T_c = 4.0$ and $\alpha = 4.0$. The figures correspond to $u = -0.5, 0.0, 0.5$ from left to right for $\kappa = -0.6$ (top) and $\kappa = 0.6$ (bottom). The Brownian magneto-gyrator operates as a microengine even in the absence of the parameter u due to the exerted torque by the particle on the potential. In this case, the direction of the gyration can be reversed by reversing the direction of the magnetic field resulting in a rotation in the probability density, shown in the middle figures. If $u \neq 0$ this can be done by additionally changing the sign of the potential coupling parameter or tuning further the applied magnetic field. The direction of the fluxes are shown by arrows and the magnitude is color coded. The right panel shows that the microengine can be stalled, which means that $\mathrm{J} = \mathbf{0}$, either if $\alpha = 1$ and $u = 0$ or $\kappa = 2u/(\alpha - 1)$ and $u \neq 0$ since the particle does not exert any torque. These cases are shown at the top and bottom, respectively. Note that the particle does not perform any gyrating motion if its motion is not correlated, namely when u and κ are zero.

Stationary-State Properties

In order to investigate the stationary-state properties of the system, we use the generalized Fokker-Planck equation corresponding to the overdamped Langevin equation (18), which can be written as

$$\frac{\partial \rho(\boldsymbol{r},t)}{\partial t} = -\nabla \cdot \mathrm{J}(\boldsymbol{r},t), \tag{19}$$

where $\rho(\boldsymbol{r},t)$ is the probability density of finding the particle at position $\boldsymbol{r}$ at time t and $\mathrm{J}(\boldsymbol{r},t)$ is the probability flux, given as

$$\mathrm{J}(\boldsymbol{r},t) = -\boldsymbol{D}\nabla\rho(\boldsymbol{r},t) - (\boldsymbol{A}\cdot\boldsymbol{r})\rho(\boldsymbol{r},t), \tag{20}$$

where the matrix $\boldsymbol{D}$ can be derived from Eq. (18)[2] or alternatively by using a first-priciple approach [3], which is given as

$$\boldsymbol{D} = \frac{1}{\gamma}\begin{pmatrix} \frac{T_c+\kappa^2 T_h}{(1+\kappa^2)^2} & \frac{\kappa(T_h-T_c)}{(1+\kappa^2)^2} + \frac{\kappa(T_c+T_h)}{2(1+\kappa^2)} \\ \frac{\kappa(T_h-T_c)}{(1+\kappa^2)^2} - \frac{\kappa(T_c+T_h)}{2(1+\kappa^2)} & \frac{T_h+\kappa^2 T_c}{(1+\kappa^2)^2} \end{pmatrix}. \tag{21}$$

Surprisingly, despite the overdamped motion of the particles, the dynamics preclude a purely diffusive description as can be seen in the unusual structure of $\boldsymbol{D}$ above. In contrast to typical diffusion tensors, $\boldsymbol{D}$ has antisymmetric elements. These give rise to additional rotational fluxes in the system, which are perpendicular to the typical diffusive fluxes[1,4–7]. One of the

known features of Lorentz forces is the reduction of the diffusion by a factor of $1/(1+\kappa^2)$, as can be seen from Eq. (21) for $T_c = T_h = T$. Note that under Lorentz force, the diffusion is spatially correlated as can be seen from the symmetric, off-diagonal terms which are non-zero only for $T_h \neq T_c$ and $\kappa \neq 0$. Throughout this work we set the Boltzmann constant k_B to unity.

Equation (19) is a linear Fokker-Planck equation whose Gaussian solution can be written as

$$\rho(\boldsymbol{r},t) = \frac{1}{Z} e^{-\frac{1}{2}(\boldsymbol{r}^\top \cdot \mathbb{M}_t^{-1} \cdot \boldsymbol{r})}, \tag{22}$$

where $Z = 2\pi\sqrt{\mathrm{Det}(\mathbb{M}_t)}$ is the normalization factor implying the probability density function with total probability of one and the covariance matrix $\mathbb{M}_t$ satisfies the following Lyapunov equation

$$\frac{\mathrm{d}\mathbb{M}_t}{\mathrm{d}t} = \boldsymbol{A}\mathbb{M}_t + \mathbb{M}_t\boldsymbol{A}^\top + 2\boldsymbol{D}_s \tag{23}$$

where $\boldsymbol{D}_s = (\boldsymbol{D}+\boldsymbol{D}^\top)/2$ is the usual (symmetric) diffusion matrix. Equation (23) can be solved for the stationary state by setting $\mathrm{d}\mathbb{M}_t/\mathrm{d}t$ to zero. This gives the stationary-state covariance matrix, denoted by $\mathbb{M}$, as

$$\mathbb{M} = \begin{pmatrix} \frac{\alpha^2(1+\kappa^2)T_c+u^2(T_h-T_c)+\alpha(T_c+\kappa^2 T_h)}{k(1+\alpha)(\alpha-u^2)(1+\kappa^2)} & -\frac{\kappa(\alpha-u^2)(T_h-T_c)-u(1+\kappa^2)(\alpha T_c+T_h)}{k(1+\alpha)(\alpha-u^2)(1+\kappa^2)} \\ -\frac{\kappa(\alpha-u^2)(T_h-T_c)-u(1+\kappa^2)(\alpha T_c+T_h)}{k(1+\alpha)(\alpha-u^2)(1+\kappa^2)} & \frac{(1+\kappa^2)T_h-u^2(T_h-T_c)+\alpha(\kappa^2 T_c+T_h)}{k(1+\alpha)(\alpha-u^2)(1+\kappa^2)} \end{pmatrix}, \tag{24}$$

The stationary-state probability density can be written as $\rho(\boldsymbol{r}) = e^{-\frac{1}{2}(\boldsymbol{r}^\top \cdot \mathbb{M}^{-1} \cdot \boldsymbol{r})}/Z$ where $\mathbb{M}^{-1}$ and Z are given as

$$Z = \frac{2\pi\sqrt{[u(T_h-T_c)+\kappa(\alpha T_c+T_h)]^2+(1+\alpha)^2 T_c T_h}}{k(1+\alpha)\sqrt{(1+\kappa^2)(\alpha-u^2)}}, \qquad \text{and} \qquad \mathbb{M}^{-1} = \begin{pmatrix} \mu_1 & \mu_3 \\ \mu_3 & \mu_2 \end{pmatrix}, \tag{25}$$

where

$$\mu_1 = k(1+\alpha)\frac{(1+\kappa^2)T_h - u^2(T_h-T_c)+\alpha(\kappa^2 T_c+T_h)]}{[u(T_h-T_c)+\kappa(\alpha T_c+T_h)]^2+(1+\alpha)^2 T_c T_h}, \tag{26}$$

$$\mu_2 = k(1+\alpha)\frac{\alpha^2(1+\kappa^2)T_c + u^2(T_h-T_c)+\alpha(T_c+\kappa^2 T_h)}{[u(T_h-T_c)+\kappa(\alpha T_c+T_h)]^2+(1+\alpha)^2 T_c T_h}, \tag{27}$$

$$\mu_3 = k(1+\alpha)\frac{u(\alpha T_c+T_h)(1+\kappa^2)-\kappa(\alpha-u^2)(T_h-T_c)}{[u(T_h-T_c)+\kappa(\alpha T_c+T_h)]^2+(1+\alpha)^2 T_c T_h}. \tag{28}$$

Note that in the case of an isotropic potential with $u = 0$ and $\alpha = 1$ Eqs. (25) to (28) reduce to the results reported in Ref.[3] . The probability fluxes in the stationary state of the system can be determined by substituting the stationary-state probability density into Eq. (20).

In the left panel of Fig. 2 we show the stationary-state probability density of the position of the particle and the fluxes in the system for different values of the diffusive Hall parameter κ and the potential coupling parameter u. The fluxes are shown on top of the probability density. The Brownian magneto-gyrator does not essentially require a positional coupling due to the potential, as shown in the middle figures for $u = 0$. However, the generic scenario in the presence of the potential coupling parameter, corresponding to $u \neq 0$, is also investigated that is shown in the left and right figures. The direction of the gyration of the microengine can be reversed by tuning the strength and direction of the applied magnetic field. In doing so, one rotates the probability density as well.

Exerted Torque

To obtain a simple scalar quantifier for the strength of the gyrating current field, which further emphasizes the tunability of the magneto-gyrator, we investigate the average torque on the potential. The average exerted torque by the particle on the potential V, denoted by $\langle\tau\rangle$, can be written as

$$\langle\tau\rangle = \int \rho(\boldsymbol{r})(\boldsymbol{r}\times\boldsymbol{F}_c)\,\mathrm{d}\boldsymbol{r}, \tag{29}$$

which is exactly equal to the opposite torque which the particle exerts via the friction forces on the thermal environment. The substitution of Eq. (22) for the stationary-state into Eq. (29) gives the average exerted torque on the potential by the particle as

$$\langle\tau\rangle = \frac{(T_h-T_c)[2u+(1-\alpha)\kappa]}{(1+\alpha)(1+\kappa^2)}. \tag{30}$$

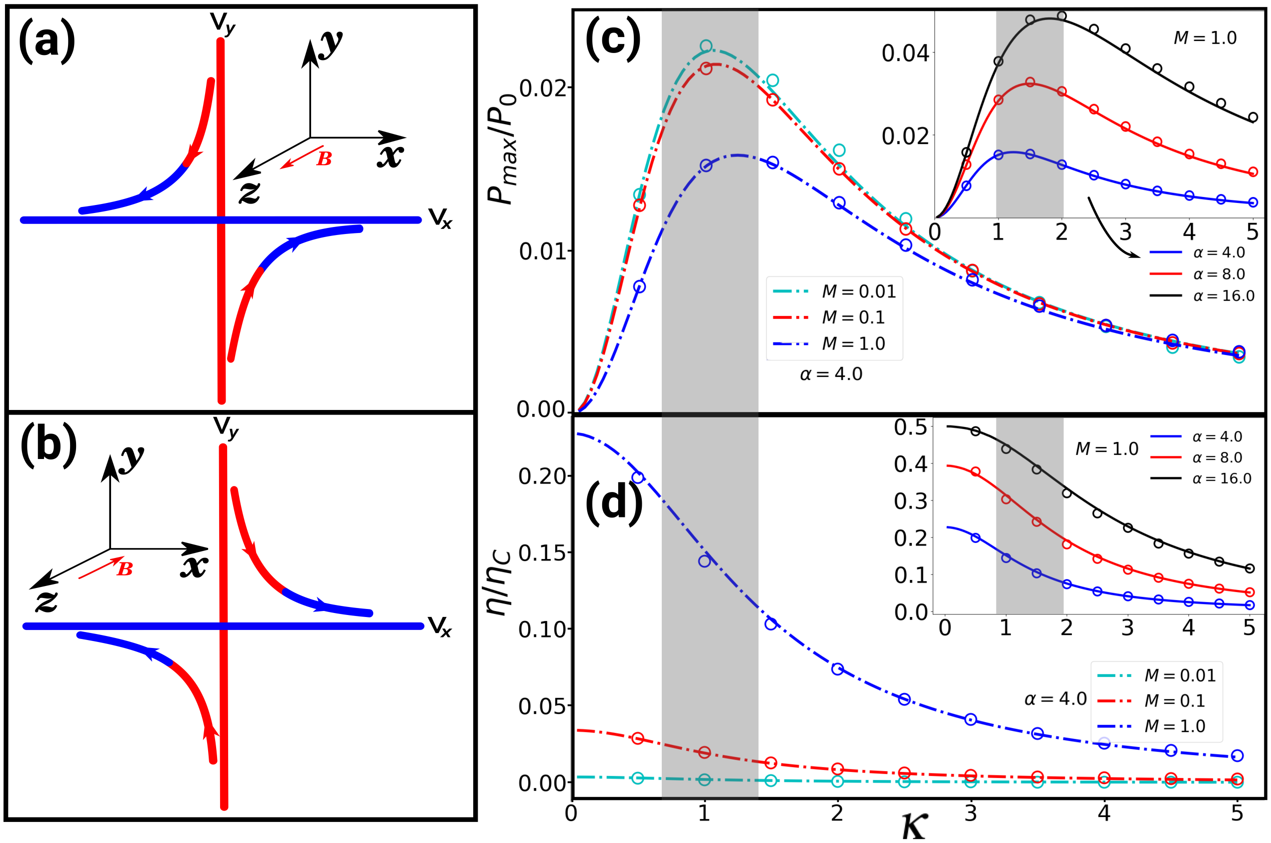

Figure 3. Average exerted torque by the microengine as a function of (a) diffusive Hall parameter κ for different values of α and (b) the parameter α for different values of κ with $T_h = 4T_c = 4.0$. The main figures show the results for $u = 0.5$ while the insets depict those for $u = 0$. As shown in (a), the average torque from Eq. (30) shows a maximum at an optimal κ, which can be reversed by tuning the strength and direction of the applied magnetic field. (b) In the generic scenario when $u \neq 0$, with increasing α the average torque increases if $\kappa < -0.5$ and decreases if $\kappa > -0.5$ till the exerted torque changes the direction resulting in the reversal of the gyration. It is independent of α if $\kappa = -0.5$. For $u = 0$ with increasing α, $\langle \tau \rangle$ increases if $\kappa < 0$, decreases if $\kappa > 0$ till the torque changes the sign, and is zero when $\kappa = 0$. In addition, there is no torque if $\alpha = 1$ and $\kappa \neq 0$, which correspond to the stalled microengine.

Note that Eq. (30) implies that even if $u = 0$ there still exists a systematic torque giving rise to a nonequilibrium stationary state carrying fluxes, corresponding to an operating magneto-gyrator. This is due to the applied magnetic field ($\kappa \neq 0$) and the asymmetry of the potential about the origin ($\alpha \neq 1$). In this case, the torque exerted by the microengine on the potential can be reversed by reversing the direction of the applied magnetic field. This results in the reversal of the gyration, which is shown in the middle figures of the left panel of Fig. 2. In the presence of the potential coupling parameter, this can be done by additionally changing the sign of the potential coupling parameter or tuning further the applied magnetic field, shown in the left and right figures of Fig. 2. The average exerted torque is zero and consequently the microengine is stalled in two cases: (i) in the absence of the potential coupling parameter if $\alpha = 1$ and (ii) if the potential is anisotropic with a nonzero u, but $\kappa = 2u/(\alpha - 1)$. The absence of fluxes in these cases are due to the cessation of the average torque which correspond to stalled microengines. The results for the two cases are shown in the right panel of Fig. 2.

In Fig. 3 (a), we show that there exists a maximum torque exerted by the microengine at an optimal magnetic field. The average torque can be reversed by reversing the direction of the magnetic field when $u = 0$, which is shown in the inset. In the presence of the potential coupling parameter, shown in the main figure, this can be done by tuning further the applied magnetic field. Figure 3 (b) shows how the exerted torque by the microengine varies by tuning the stiffness of the potential for different values of the diffusive Hall parameter. The main figure represents the results for the Brownian magneto-gyrator without the

potential coupling parameter while those for the generic scenario, i.e., in the presence of u, are shown in the inset. From Eq. (30) it is clear that the average torque is independent of the parameter α if $u+\kappa=0$, which is shown in the main panel. The exerted torque decreases (increases) with increasing α if $\kappa>-u$ ($\kappa<-u$) till it changes the direction, corresponding to the change of the direction of the gyration. A similar explanation holds when $u=0$. However, there is no torque if $\kappa=0$ or $\alpha=1$ and consequently the microengine is stalled.

Mechanical Power

Now we consider the microengine in the presence of a load in order to determine the delivered mechanical power by the engine. To do this, we apply a linear external nonconservative force ($\nabla\times\boldsymbol{F}_{nc}\neq\boldsymbol{0}$) of the form $\boldsymbol{F}_{nc}=\varepsilon(-y,x)^\top$ with a parameter ε, yielding a torque in the z direction, whose sign is chosen such that the resulting torque in the z direction is opposed to $\langle\tau\rangle$ in Eq. (30). The goal is to calculate the average power of the work done by this force. The average extracted mechanical power $P=-\langle\boldsymbol{F}_{nc}^\top.\dot{\boldsymbol{r}}\rangle$ in the stationary state can be rewritten as $P=-\varepsilon\langle xv_y-yv_x\rangle$. Therefore, one needs to calculate the stationary-state position-velocity correlation matrix, that is $\lim_{t\to\infty}\langle\boldsymbol{r}(t)\dot{\boldsymbol{r}}^\top(t)\rangle$. Under this condition, the motion of a particle with mass m and velocity $\boldsymbol{v}=(v_x,v_y)^\top$ can be described by the generalised Langevin equation written as $m\dot{\boldsymbol{v}}=-\boldsymbol{G}\cdot\boldsymbol{v}-\nabla V(\boldsymbol{r})+\boldsymbol{F}_{nc}+\boldsymbol{\xi}(t)$ where $\boldsymbol{\xi}(t)=(\xi_x,\xi_y)^\top$ is Gaussian white noise with zero mean and time correlation $\langle\boldsymbol{\xi}(t)\boldsymbol{\xi}^\top(t')\rangle=2\gamma\boldsymbol{diag}(T_c,T_h)\delta(t-t')$ where $\boldsymbol{diag}(T_c,T_h)$ is a diagonal matrix. In this case, the conservative and nonconservative forces can be combined and the overdamped Langevin equation can be written in the form of Eq. (18) with a modified matrix U_l under loading, defined as

$$\mathrm{U}_l=k\begin{pmatrix}1 & u+\varepsilon'\\ u-\varepsilon' & \alpha\end{pmatrix}, \tag{31}$$

where $\varepsilon'=\varepsilon/k$ is a dimensionless parameter. Using the equation of motion in Eq. (18) it can be shown that

$$\langle\boldsymbol{r}\dot{\boldsymbol{r}}^\top\rangle=-\langle\boldsymbol{r}\boldsymbol{r}^\top\rangle\boldsymbol{A}_l^\top+\langle\boldsymbol{r}\boldsymbol{\chi}^\top\rangle, \tag{32}$$

where $\boldsymbol{A}_l=\boldsymbol{G}^{-1}\mathrm{U}_l$. Note that by applying a prescribed external vortex flow field such as a rotating bucket to an underdamped Brownian particle one can induce similar terms to the nonconservative force in the overdamped limit[8]. Moreover, it is known that in experiments using optical tweezers, optical scattering forces actually generate a nonconservative component to the overall force exerted by the trap and do not simply furnish a simple potential[9]. Here we take a linear nonconservative force for the convenience of analytical calculations. The position correlation and the position-noise correlation can be written as

$$\langle\boldsymbol{r}\boldsymbol{r}^\top\rangle = \lim_{t\to\infty}\int_0^t \mathrm{d}t'\int_0^t \mathrm{d}t'' e^{-\boldsymbol{A}_l(t-t')}\langle\boldsymbol{\chi}(t')\boldsymbol{\chi}^\top(t'')\rangle e^{-\boldsymbol{A}_l^\top(t-t'')}, \tag{33}$$

$$\langle\boldsymbol{r}\boldsymbol{\chi}^\top\rangle = \lim_{t\to\infty}\int_0^t \mathrm{d}t' e^{-\boldsymbol{A}_l(t-t')}\langle\boldsymbol{\chi}(t')\boldsymbol{\chi}^\top(t)\rangle. \tag{34}$$

The substitution of the properties of the Gaussian nonwhite noise from Eq. (**??**) into Eq. (33) and Eq. (34) gives

$$\langle\boldsymbol{r}\boldsymbol{r}^\top\rangle = \int_0^\infty \mathrm{d}s e^{-\boldsymbol{A}_l s}[\boldsymbol{G}^{-1}\boldsymbol{C}+\boldsymbol{C}(\boldsymbol{G}^{-1})^\top]e^{-\boldsymbol{A}_l^\top s}, \tag{35}$$

$$\langle\boldsymbol{r}\boldsymbol{\chi}^\top\rangle = \boldsymbol{C}(\boldsymbol{G}^{-1})^\top, \tag{36}$$

where Eq. (35) is the solution to the following Lyapunov equation

$$\boldsymbol{A}_l\langle\boldsymbol{r}\boldsymbol{r}^\top\rangle+\langle\boldsymbol{r}\boldsymbol{r}^\top\rangle\boldsymbol{A}_l^\top=-\Xi, \tag{37}$$

where $\Xi=\boldsymbol{G}^{-1}\boldsymbol{C}+\boldsymbol{C}(\boldsymbol{G}^{-1})^\top$. The stationary-state position-velocity matrix can be calculated and written as

$$\langle\boldsymbol{r}\dot{\boldsymbol{r}}^\top\rangle=\begin{pmatrix}0 & \frac{[u-\frac{\kappa}{2}(\alpha-1)](T_h-T_c)+\varepsilon'(1+\kappa^2)(T_c+T_h)}{\gamma(1+\kappa^2)(1+\alpha-2\varepsilon'\kappa)}\\ -\frac{[u-\frac{\kappa}{2}(\alpha-1)](T_h-T_c)+\varepsilon'(1+\kappa^2)(T_c+T_h)}{\gamma(1+\kappa^2)(1+\alpha-2\varepsilon'\kappa)} & 0\end{pmatrix}, \tag{38}$$

and the average mechanical power reads

$$P=2k\varepsilon'\frac{[u-\frac{\kappa}{2}(\alpha-1)](T_h-T_c)+\varepsilon'(1+\kappa^2)(T_c+T_h)}{\gamma(1+\kappa^2)(1+\alpha-2\varepsilon'\kappa)}, \tag{39}$$

which can be rewritten as

$$P=\frac{h}{1-\lambda\frac{\varepsilon}{\varepsilon_s}}\frac{\varepsilon}{\varepsilon_s}\left(1-\frac{\varepsilon}{\varepsilon_s}\right), \tag{40}$$

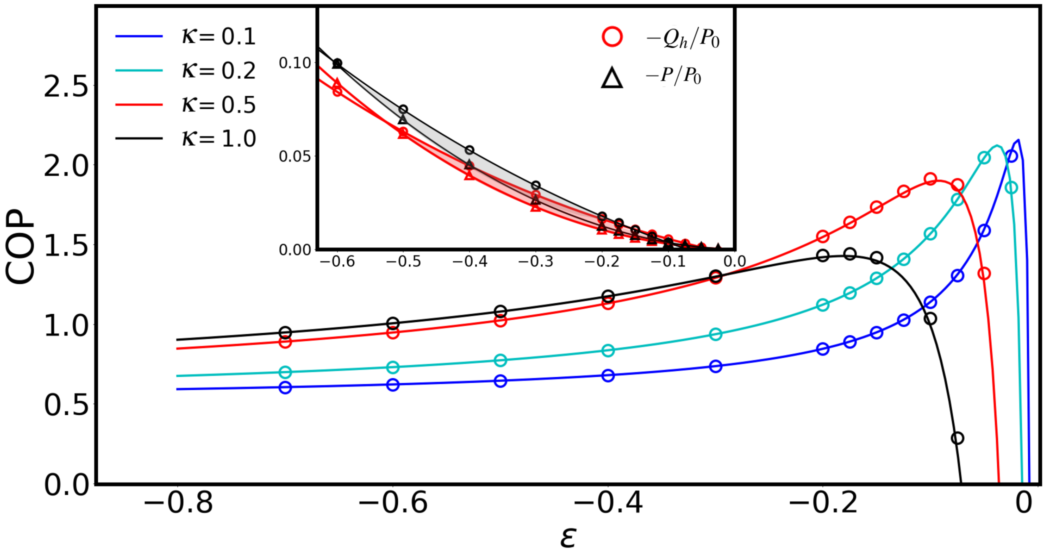

Figure 4. The power of a Brownian magneto-gyrator with $T_h = 4T_c = 4.0$ and $u = 0.0$. The main panel shows the scaled power P/P_{max} as a function of $\varepsilon/\varepsilon_s$. The mechanical power vanishes when ε is zero or equal to the stall value. In addition, it has a maximum at $\varepsilon/\varepsilon_s = (1-\sqrt{1-\lambda})/\lambda$ where $\lambda = 2\kappa\varepsilon_s/(k(1+\alpha))$ which can be shifted by tuning the magnetic field. For the upper inset we use Eq. (41) to plot the stall parameter with respect to the diffusive Hall parameter with $\alpha = 4.0$. The stall parameter approaches zero with increasing magnitude of the applied magnetic field. In the lower inset, we show how the maximum power varies with respect to the diffusive Hall parameter for different values of α. P_{max} is plotted in units of $P_0 = k(T_c+T_h)/\gamma$. There is an optimal value of κ for which the maximum power takes its highest value. The lines show the theoretical predictions and the symbols are simulation results.

where $h = 2\varepsilon_s^2(T_c+T_h)/(k\gamma(1+\alpha))$, $\lambda = 2\kappa\varepsilon_s/(k(1+\alpha))$, and ε_s is the stall parameter which quantifies the maximum strength of the nonconservative force that one can apply to the engine without driving it in the inverse direction. Hence, the system operates as a heat engine, delivering mechanical work for $0 < \frac{\varepsilon}{\varepsilon_s} < 1$. The stall parameter is given as

$$\varepsilon_s = -k\frac{\left(u-\frac{\kappa}{2}(\alpha-1)\right)\eta_c}{(2-\eta_c)(1+\kappa^2)}, \tag{41}$$

where $\eta_c = 1 - T_c/T_h$ is the Carnot efficiency. Having demonstrated in details the tunability of a Brownian magneto-gyrator in the presence of a rotated anisotropic potential, we focus from this point onwards on pure magneto-gyration, setting $u = 0$.

The main panel of Fig. 4 shows the scaled power P/P_{max} delivered by the magneto-gyrator as a function of scaled loading $\varepsilon/\varepsilon_s$. As expected, the mechanical power vanishes when the engine is unloaded, i.e., $\varepsilon = 0$ or when the engine is stalled at $\varepsilon = \varepsilon_s$. The engine operates at maximum power at an intermediate loading $\varepsilon/\varepsilon_s = (1-\sqrt{1-\lambda})/\lambda$ which can be shifted by tuning the applied magnetic field. As can be seen in the inset to Fig. 4, there exists an optimal value of κ for which the microengine delivers the maximum mechanical power. Qualitatively, this can be understood as follows. In the limit of vanishing magnetic field, there is no spatial correlation and hence no gyration. In the opposite case of very large magnetic field, the correlations vanish due to the large reduction in the diffusion coefficient of the particle.

Efficiency of a Mesoscopic Brownian Heat Engine

In this section, we calculate the stationary-state covariance matrix whose elements are needed to measure the efficiency of the heat engine and the COP of a magneto-gyrator. The Langevin equation (1) for the particle under a load of the form of the nonconservative force $\boldsymbol{F}_{nc} = \varepsilon(-y,x)^\top$ with the parameter ε and in the harmonic potential $\mathrm{V}(x,y) = k(x^2+\alpha y^2)/2$ can be rewritten as

$$\dot{\boldsymbol{z}}(t) = -\boldsymbol{F}\boldsymbol{z}(t) + \tilde{\boldsymbol{\xi}}(t), \tag{42}$$

where $\boldsymbol{z}(t) = (x(t),y(t),v_x(t),v_y(t))^\top$ and $\tilde{\boldsymbol{\xi}}(t) = (0,0,m^{-1}\xi_x(t),m^{-1}\xi_y(t))^\top$ is Gaussian white noise with zero mean and time correlation $\langle\tilde{\boldsymbol{\xi}}(t)\tilde{\boldsymbol{\xi}}^\top(t')\rangle = (2\gamma/m^2)\mathrm{T}\delta(t-t')$ where γ is the constant friction coefficient. Here k is the stiffness of the potential,

α is a dimensionless parameter, and $\mathrm{T} = \boldsymbol{diag}(0,0,T_c,T_h)$ is a diagonal matrix. Note that T is a two-dimensional matrix in Eq. (1). The matrix $\boldsymbol{F}$ is defined as

$$\boldsymbol{F} = \frac{1}{m}\begin{pmatrix} \mathbf{0} & -m\mathbb{I} \\ \mathrm{U}_l & \boldsymbol{G} \end{pmatrix}, \tag{43}$$

where $\mathbb{I}$ is the identity matrix and

$$\boldsymbol{G} = \gamma\begin{pmatrix} 1 & -\kappa \\ \kappa & 1 \end{pmatrix}, \qquad \mathrm{U}_l = k\begin{pmatrix} 1 & \varepsilon' \\ -\varepsilon' & \alpha \end{pmatrix}. \tag{44}$$

where $\varepsilon' = \varepsilon/k$ is a dimensionless parameter. In order to calculate the efficiency of the microengine the calculation of the average rate of the heat out of the hot bath, $\langle đQ_c/dt\rangle$ and the average mechanical power, P is needed, which can be determined by the steady-state covariance matrix $\mathrm{S} = \lim_{t\to\infty}\mathrm{S}(t)$, where $\mathrm{S}(t) = \langle \boldsymbol{z}(t)\boldsymbol{z}^\top(t)\rangle$. The change in the covariance matrix in the time interval $\mathrm{d}t$ is given by

$$\mathrm{dS}(t) = -[\boldsymbol{F}\mathrm{S}(t) + \mathrm{S}(t)\boldsymbol{F}^\top]\,\mathrm{d}t + \int_t^{t+\mathrm{d}t}\mathrm{d}t'\int_t^{t+\mathrm{d}t}\mathrm{d}t''\langle\tilde{\boldsymbol{\xi}}(t')\tilde{\boldsymbol{\xi}}^\top(t'')\rangle, \tag{45}$$

which using the property of the noise $\tilde{\boldsymbol{\xi}}$, the time evolution of the covariance matrix can be written as

$$\frac{\mathrm{dS}(t)}{\mathrm{d}t} = -\boldsymbol{F}\mathrm{S}(t) - \mathrm{S}(t)\boldsymbol{F}^\top + \frac{2\gamma}{m^2}\mathrm{T}. \tag{46}$$

The steady-state covariance matrix can be calculated by setting $\mathrm{dS}(t)/\mathrm{d}t$ to zero, which consists of the steady-state position-position, position- and velocity-velocity correlations. The solution to Eq. (46) gives the steady-state covarinace matrix with the following elements

$$\begin{aligned}
\langle x^2\rangle &= \frac{[(\alpha-\alpha^2+\varepsilon'^2)(1-\alpha-2\varepsilon'\kappa)-2\alpha\varepsilon'^2]M + 2[\varepsilon'^2+(1+\kappa^2)\alpha^2+\alpha]}{S_0 k(\varepsilon'^2+\alpha)}T_c \\
&+ \frac{\varepsilon'^2(1+\alpha+2\varepsilon'\kappa)M + 2(1+\kappa^2)\varepsilon'^2 + 2\alpha\kappa^2}{S_0 k(\varepsilon'^2+\alpha)}T_h, && (47)\\
\langle y^2\rangle &= \frac{\varepsilon'^2(1+\alpha+2\varepsilon'\kappa)M + (1+\kappa^2)\varepsilon'^2 + \alpha\kappa^2}{S_0 k(\varepsilon'^2+\alpha)}T_c \\
&+ \frac{[(1-\alpha^2)-\varepsilon'(1-\alpha)(\varepsilon'-2\varepsilon'\kappa)-2\varepsilon'^2(1+\varepsilon'\kappa)]M + 2(1+\alpha+\varepsilon'^2+\kappa^2)}{S_0 k(\varepsilon'^2+\alpha)}T_h, && (48)\\
\langle v_x^2\rangle &= k\frac{[(\alpha-1)^2-2\varepsilon'(\varepsilon'+(1-\alpha)\kappa)]M + (2+\kappa^2)(1+\alpha-2\varepsilon'\kappa)}{\gamma^2 M S_0}T_c + k\frac{\kappa^2(1+\alpha-2\varepsilon'\kappa)+2\varepsilon'^2 M}{\gamma^2 M S_0}T_h, && (49)\\
\langle v_y^2\rangle &= k\frac{\kappa^2(1+\alpha-2\varepsilon'\kappa)+2\varepsilon'^2 M}{\gamma^2 M S_0}T_c + k\frac{[(\alpha-1)^2-2\varepsilon'(\varepsilon'-(1-\alpha)\kappa)]M + (2+\kappa^2)(1+\alpha-2\varepsilon'\kappa)}{\gamma^2 M S_0}T_h, && (50)\\
& && (51)
\end{aligned}$$

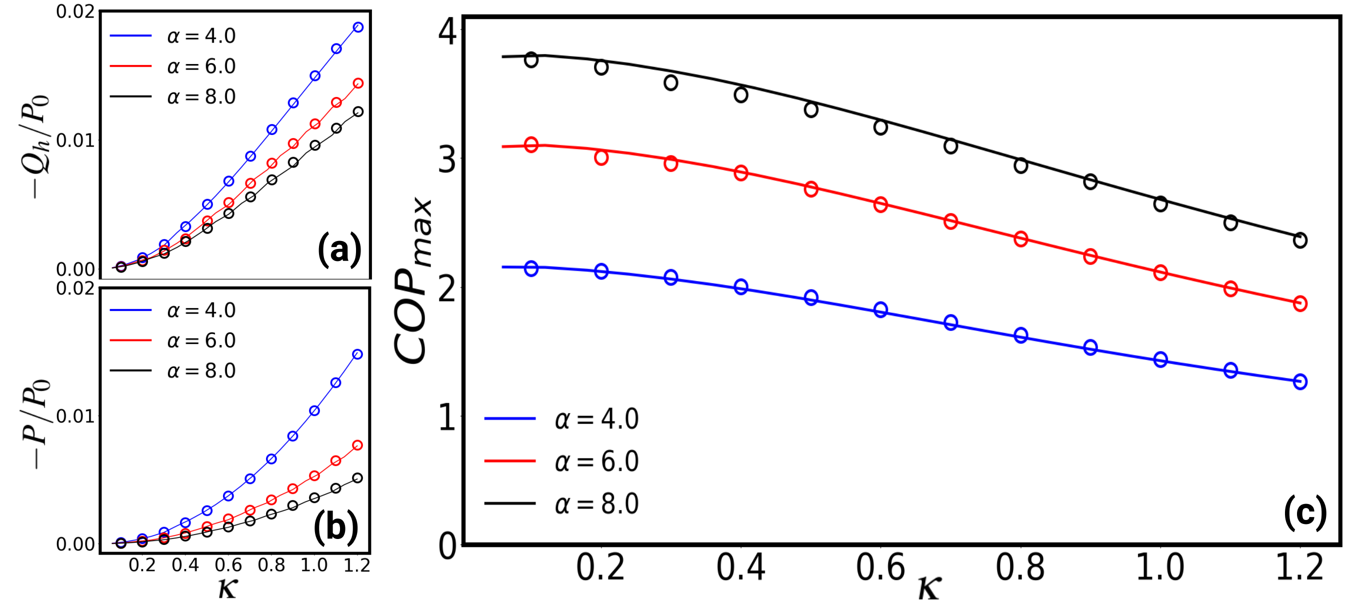

Figure 5. The stationary-state probability density of the particle's position and fluxes in a system with $M = 1.0$, $\alpha = 4.0$, $\kappa = 1.0$, $u = 0.0$, and $T_h = 4T_c = 4.0$. The results are shown from Brownian dynamics simulations to confirm the stability of the microengine for $\varepsilon = 0.0$, $\varepsilon < \varepsilon_s$, and the stalled engine $\varepsilon = \varepsilon_s$ from left to right, respectively.

where $M = km/\gamma^2$ is a dimensionless parameter and the non-diagonal elements are given as

$$\begin{aligned}
\langle xy\rangle &= \frac{[\alpha(\alpha-1+2\varepsilon'\kappa)-2\varepsilon'^2]\varepsilon' M+2[\alpha(\varepsilon'-\kappa+\varepsilon'\kappa^2)-\varepsilon'^2]}{S_0 k(\varepsilon'^2+\alpha)}T_c \\
&+ \frac{(2\varepsilon'^2+\alpha-1-2\varepsilon'\kappa)\varepsilon' M+2[\kappa(\alpha+\varepsilon'^2)-\varepsilon'(1+\kappa^2)]}{S_0 k(\varepsilon'^2+\alpha)}T_h, && (52)\\
\langle yx\rangle &= \langle xy\rangle, && (53)\\
\langle xv_x\rangle &= \langle v_x x\rangle = 0, && (54)\\
\langle yv_y\rangle &= \langle v_y y\rangle = 0, && (55)\\
\langle xv_y\rangle &= -\frac{\kappa(1-\alpha)(T_h-T_c)+2\varepsilon'(1+\kappa^2)(T_c+T_h)}{S_0}, && (56)\\
\langle v_y x\rangle &= \langle xv_y\rangle, && (57)\\
\langle yv_x\rangle &= -\langle xv_y\rangle, && (58)\\
\langle v_x y\rangle &= \langle yv_x\rangle, && (59)\\
\langle v_x v_y\rangle &= k\frac{[(\alpha-1)(T_c+T_h)-2\kappa\varepsilon'^2(T_h-T_c)]\varepsilon' M+\kappa(1+\alpha-2\varepsilon'\kappa)(T_h-T_c)}{\gamma M S_0}, && (60)\\
\langle v_y v_x\rangle &= \langle v_x v_y\rangle, && (61)\\
& && (62)
\end{aligned}$$

where $S_0 = [(\alpha-1)^2-4\varepsilon'^2(1+\kappa^2)]M+2(1+\kappa^2)(1+\alpha-2\varepsilon'\kappa)$. Note that taking the small-mass limit of the covariance matrix gives the correlations in the overdamped regime. Since we finally need the kinetic energy of the particle which is proportional to $M(\langle v_x^2\rangle+\langle v_y^2\rangle)$, in the overdamped regime the small-mass limit of $M\langle v_x^2\rangle$ and $M\langle v_y^2\rangle$ is required which makes Eq. (49) and Eq. (50) well-defined. However, for the cross-correlation in Eq. (60) the explicit knowledge of the particle's mass is needed even in the overdamped limit.

The efficiency is given by the ratio between the average rate of heat extracted from the hot bath and the total energy transferred. The average rate of the heat out of the hot bath, $Q_h = \gamma\kappa\langle v_x v_y\rangle - \varepsilon\langle xv_y - yv_x\rangle/2$, can be calculated using Eq. (60) which reads as $Q_h = f(\kappa, M, \varepsilon)/M$ where $Q_h = \langle đQ_h/dt\rangle$ and

$$f(\kappa,M,\varepsilon) = k\frac{[(\kappa(\alpha-1)+2\varepsilon'(1+\kappa^2))(T_c+T_h)+\kappa(1-\alpha-2\kappa\varepsilon'^2)(T_h-T_c)]\varepsilon' M+\kappa^2(1+\alpha-2\varepsilon'\kappa)(T_h-T_c)}{\gamma S_0}, \quad (63)$$

and similarly the average mechanical power, $P = -\varepsilon\langle xv_y - yv_x\rangle$, can be written as

$$P = 2k\varepsilon' \frac{\kappa(1-\alpha)(T_h - T_c) + 2\varepsilon'(1+\kappa^2)(T_c + T_h)}{\gamma S_0}. \tag{64}$$

The efficiency of the magneto-gyrator, defined as $\eta = P/Q_h$, can be calculated and written as

$$\eta = \frac{2\varepsilon' M[\kappa(1-\alpha)\eta_c + 2\varepsilon'(1+\kappa^2)(2-\eta_c)]}{\varepsilon' M[(\kappa(\alpha-1) + 2\varepsilon'(1+\kappa^2))(2-\eta_c) + \kappa(1-\alpha-2\kappa\varepsilon'^2)\eta_c] + \kappa^2(1+\alpha-2\varepsilon'\kappa)\eta_c}, \tag{65}$$

where $\eta_c = 1 - T_c/T_h$ is the Carnot efficiency. The efficiency as a function of the diffusive Hall parameter is plotted in Fig. 3 (d) in the main text.

The existence condition of the steady state can be found by investigating the stability condition, which for our proposed magneto-gyrator can be done via studying the divergence of mechanical power in Eq.(64). This leads to $S_0 > 0$, which can be written as

$$[(\alpha-1)^2 - 4\varepsilon'^2(1+\kappa^2)]M + 2(1+\kappa^2)(1+\alpha-2\varepsilon'\kappa) > 0, \tag{66}$$

which in the case of $\alpha = 1.0$ reduces to $1 + \varepsilon'\kappa - M\varepsilon'^2 > 0$ which has been reported in Ref.[11]. In Fig. 5 we show the stationary-state probability density of the particle's position and fluxes in a system with the suggested parameters for the possible experimental realisation in the main text. The simulation results are in agreement with our theoretical stability condition and confirm that the magneto-gyrator is stable for all chosen parameters in this work.

COP of a Brownian Magneto-Refrigerator

A Brownian magneto-gyrator can be operated as a refrigerator: by applying the nonconservative force $\boldsymbol{F}_{nc} = \varepsilon(-y,x)^\top$ to the system with $\varepsilon < 0$. The sign of the parameter ε is chosen such that the resulting torque in the z direction is in favour of the existed torque by the Lorentz force, namely we enhance the gyration along the direction of the unloaded magneto-refrigerator. Moreover, we operate the refrigerator in a range of ε where the system is stable. By doing so, we extract heat from the cold bath and pump it into the hot bath. We obtain the coefficient of performance (COP) of refrigerator by dividing the average rate of the heat extracted from the hot bath by the corresponding input work Q_h/P, given in Eq. (63) and Eq. (64), respectively. Therefore, the COP of the magneto-refrigerator can be written as

$$\mathrm{COP} = \frac{\varepsilon' M[(\kappa(\alpha-1) + 2\varepsilon'(1+\kappa^2))(T_c+T_h) + \kappa(1-\alpha-2\kappa\varepsilon'^2)(T_h-T_c)] + \kappa^2(1+\alpha-2\varepsilon'\kappa)(T_h-T_c)}{2\varepsilon' M[\kappa(1-\alpha)(T_h-T_c) + 2\varepsilon'(1+\kappa^2)(T_c+T_h)]}. \tag{67}$$

The COP as a function of the parameter ε is plotted in Fig. 4 in the main text. The COP has a maximum at an optimal ε which is presented in Fig. 5 in the main text, as well.

References

1. Vuijk, H. D., Brader, J. M. & Sharma, A. Anomalous fluxes in overdamped Brownian dynamics with Lorentz force. *J. Stat. Mech. Theory Exp.* **2019**, 063203 (2019).
2. Chun, H.-M., Durang, X. & Noh, J. D. Emergence of nonwhite noise in Langevin dynamics with magnetic Lorentz force. *Phys. Rev. E* **97**, 032117 (2018).
3. Abdoli, I. *et al.* Correlations in multithermostat Brownian systems with Lorentz force. *New J. Phys.* **22**, 093057 (2020).
4. Abdoli, I. & Sharma, A. Stochastic resetting of active Brownian particles with Lorentz force. *Soft Matter* **17**, 1307–1316 (2021).
5. Vuijk, H. D., Brader, J. M. & Sharma, A. Effect of anisotropic diffusion on spinodal decomposition. *Soft Matter* **15**, 1319–1326 (2019).
6. Abdoli, I., Vuijk, H. D., Sommer, J.-U., Brader, J. M. & Sharma, A. Nondiffusive fluxes in a Brownian system with Lorentz force. *Phys. Rev. E* **101**, 012120 (2020).
7. Abdoli, I. *et al.* Stationary state in Brownian systems with Lorentz force. *Phys. Rev. Res.* **2**, 023381 (2020).
8. Liebchen, B. & Löwen, H. Optimal navigation strategies for active particles. *EPL (Europhysics Lett.* **127**, 34003 (2019).
9. Mangeat, M., Amarouchene, Y., Louyer, Y., Guérin, T. & Dean, D. S. Role of nonconservative scattering forces and damping on Brownian particles in optical traps. *Phys. Rev. E* **99**, 052107 (2019).

10. Seifert, U. Stochastic thermodynamics, fluctuation theorems and molecular machines. *Reports on Prog. Phys.* **75**, 126001 (2012).

11. Lee, S. & Kwon, C. Nonequilibrium driven by an external torque in the presence of a magnetic field. *Phys. Rev. E* **99**, 052142 (2019).
